# Tempo of the Late Ordovician mass extinction controlled by the rate of climate change

**DOI:** 10.1126/sciadv.adv6788

**Published:** 2025-05-30

**Authors:** Zhutong Zhang, Chuan Yang, Diana Sahy, Ren-bin Zhan, Rong-Chang Wu, Yang Li, Yiying Deng, Bing Huang, Daniel J. Condon, Jiayu Rong, Xian-Hua Li

**Affiliations:** ^1^ State Key Laboratory of Lithospheric and Environmental Coevolution, Institute of Geology and Geophysics, Chinese Academy of Sciences, Beijing 100029, China.; ^2^ College of Earth and Planetary Science, University of Chinese Academy of Sciences, Beijing 100049, China.; ^3^ Department of Earth Sciences, University College London, London WC1E 6BT, UK.; ^4^ Geochronology and Tracers Facility, British Geological Survey, Keyworth NG12 5GG, UK.; ^5^ State Key Laboratory of Palaeobiology and Stratigraphy, Nanjing Institute of Geology and Palaeontology, Chinese Academy of Sciences, Nanjing 210008, China.; ^6^ Ministry of Education Key Laboratory of Orogenic Belts and Crustal Evolution, School of Earth and Space Sciences, Peking University, Beijing 100871, China.; ^7^ School of Resources and Environmental Engineering, Hefei University of Technology, Hefei 230009, China.

## Abstract

The Late Ordovician mass extinction (LOME) included two phases (I and II) of high species turnover that have been hypothetically linked to the Hirnantian glaciation and subsequent rapid warming, respectively. However, the timing and tempo of the LOME remain uncertain, which hinders our understanding of the feedback between the LOME and paleoclimatic change. Here, we present high-precision radioisotopic dates for the Ordovician-Silurian transition in South China that reveal the LOME began at 442.76 + 0.35/−0.22 million years ago, with the two phases lasting for 0.34 + 0.46/−0.34 and 0.06 + 0.31/−0.06 million years, respectively. The rapid switch from icehouse to greenhouse conditions, along with the higher mean rate of temperature change during LOME II, resulted in a much higher mean extinction rate during LOME II than I (71.6% versus 8.4% species loss per 100 thousand years, respectively), implying that the rate of climate change was a primary control on the tempo of the LOME.

## Teaser

U-Pb geochronology refines the Ordovician-Silurian transition timescale and constrains the tempo and driving forces of the LOME.

## INTRODUCTION

The Late Ordovician mass extinction (LOME) was the earliest of the “Big Five” Phanerozoic mass extinction events (*1*, *2*). Previous studies have suggested that this event led to the extinction of ~85% of marine species, making it the second most severe biotic crisis in the Phanerozoic in terms of species loss (*1*, *3*–*5*). However, recent studies indicate that 69.7% of marine species became extinct in the latest Ordovician (*6*). This event occurred in a time interval when Earth experienced marked environmental perturbations, including the Hirnantian glaciation (*7*–*10*), increased volcanic activity (*11*–*13*), expanded marine anoxia (*12*, *14*, *15*), and true polar wander (*16*), leading to hypotheses that there were causal linkages between these factors. Furthermore, climate change is a key control on biodiversity (*17*–*20*). The different response patterns of biodiversity to climate change in the Phanerozoic indicate that the fundamental drivers of the Late Ordovician biodiversity loss may not have been simply due to the changes of environmental factors such as temperature and atmospheric CO_2_ concentrations but rather the rate of such changes (*21*, *22*).

Although a temporal link between climate change and the LOME has been well established (*9*), the timing and rate of the LOME and major climatic changes in the latest Ordovician have not yet been determined because of the lack of high-precision radioisotopic dates for the Ordovician-Silurian transition. This hinders a better understanding of the nature and interrelationships of these events. The duration of the LOME has been proposed to range from 0.98 to 2.2 million years (Myr), based largely on ion probe and multigrain zircon isotope dilution–thermal ionization mass spectrometry (ID-TIMS) U-Pb dating and cyclostratigraphy (*6*, *23*–*25*). High-precision zircon U-Pb dates for South China obtained using chemical abrasion (CA)–ID-TIMS suggest that the duration of the LOME was much shorter (<0.2 Myr) than previously thought (*26*). However, this remains controversial (*27*) and is still unresolved because of the lack of reliable high-precision radioisotopic ages from sections with robust biostratigraphic constraints. In addition, the LOME has generally been separated into two phases. The first (LOME I) affected nekton and plankton near the base of the *Diceratograptus mirus* biozone during the Katian-Hirnantian transition (*28*–*30*). The second (LOME II) affected the cool water–adapted Hirnantian fauna along with many other open marine fauna in the *Metabolograptus persculptus* biozone during the latest Hirnantian (*31*, *32*). However, these two phases have not been distinguished in the long-term trends in Ordovician biodiversity (*6*, *33*), possibly due to similar extinction rates in the two phases or that the LOME was a single-phase event (*34*). Hence, the tempo of the LOME and its cause remains unclear.

**Fig. 1.**
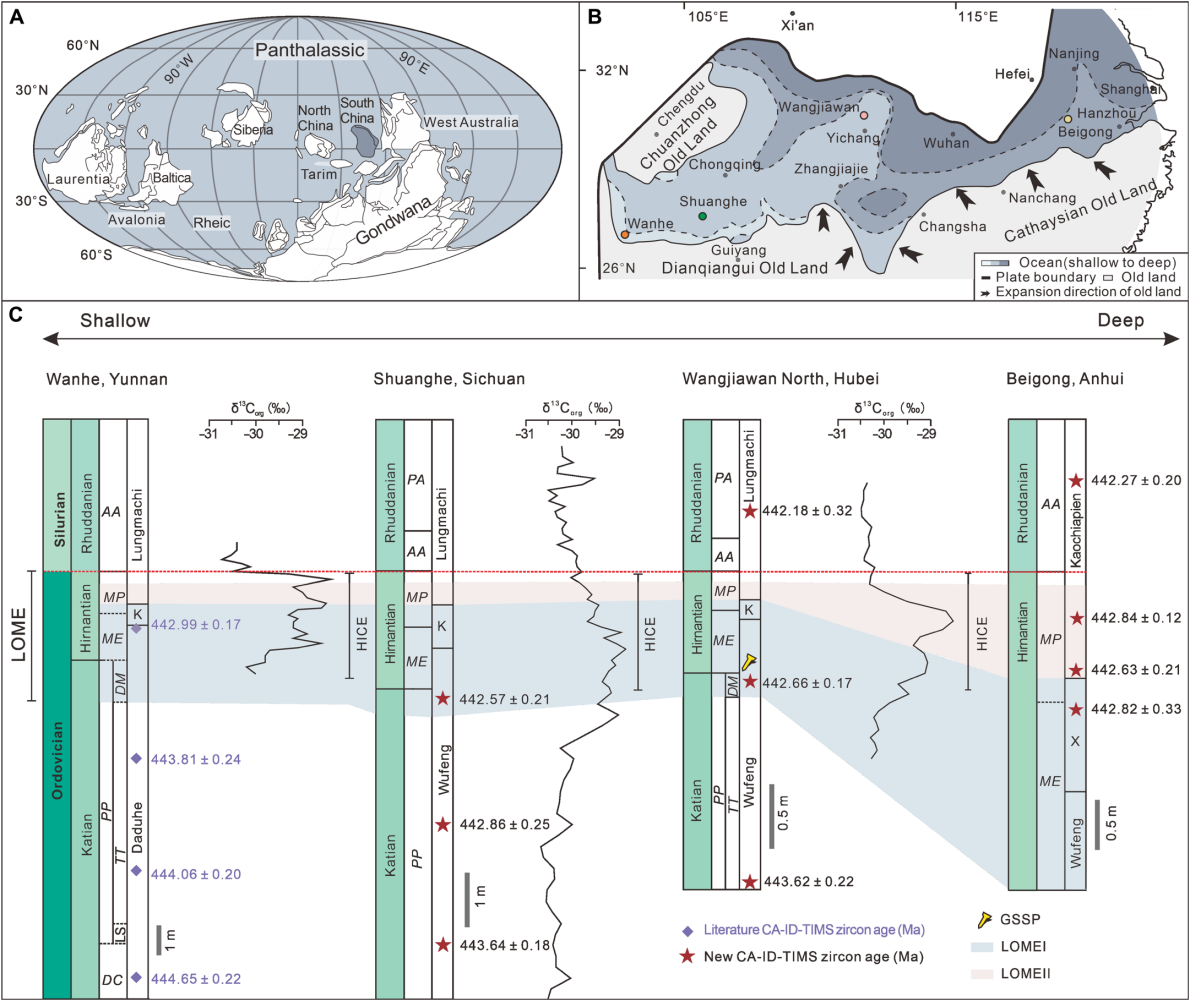
Late Ordovician paleogeographic maps and Ordovician-Silurian transitional successions of South China block. (**A**) Paleogeographic map showing the location of South China during the Late Ordovician [445 Ma; modified from figure 6B in (*69*); https://creativecommons.org/licenses/by/4.0/]. (**B**) Late Ordovician paleogeographic map of the Yangtze Platform in South China, showing the locations of the Wangjiawan North, Shuanghe, Beigong, and Wanhe sections [modified from (*29*, *70*)]. (**C**) Ordovician-Silurian transitional successions in South China [modified from (*35*, *71*)]. Published radioisotopic dates and carbon isotopic data are from (*26*, *35*, *72*) [(*26*), https://creativecommons.org/licenses/by-nc-nd/4.0/; (*35*), http://creativecommons.org/licenses/by-nc/4.0]. *PA*, *Parakidograptus acuminatus*; *AA*, *Akidograptus ascensus*; *MP*, *M. persculptus*; *ME*, *Metabolograptus extraordinarius*; *PP*, *Paraorthograptus pacificus*; *DM*, *Dicellograptus mirus*; *TT*, *Tangyagraptus typicus*; LS, lower subzone; *DC*, *Dicellograptus complexus*; K, Kuanyinchiao; X, Xinkailing.

Here, we present a high-precision geochronology of the LOME using the CA-ID-TIMS zircon U-Pb dating method. The 10 dated ash beds were collected from three Ordovician-Silurian sections along a proximal-to-distal transect in the Yangtze Block of South China (Fig. 1 and see the Supplementary Materials). Integrating these dating results with biostratigraphic, C isotopic, and biodiversity datasets, we provide a refined timescale for the Ordovician-Silurian transition. This enables us to determine the timing and rate of the LOME and major climatic changes during this time interval and constrain the potential causal linkages between the tempo of the LOME and rate of climatic change.

## RESULTS

Ten samples of ash beds were collected from the Wangjiawan North section [the Global Stratotype Section and Point (GSSP) for the Hirnantian Stage (*35*)], Shuanghe section, and Beigong section, deposited in middle shelf to slope settings, for zircon U-Pb dating (Fig. 1). Details of the studied sections in South China and organic C isotope data are given in the Supplementary Materials (figs. S1 to S3 and table S1). Seventy-two zircon grains were photographed (fig. S4) and dated by CA-ID-TIMS at the British Geological Survey. The dating results are summarized in table S2. The complete U-Pb data table is provided in the Supplementary Materials (table S3). To refine the timescale of the Ordovician-Silurian transition, we used the Bayesian Markov Chain Monte Carlo (MCMC) model in the Chron.jl package (*36*), with the weighted-mean ^206^Pb/^238^U dates used as age inputs to construct age-depth models and estimate the ages of key boundaries (figs. S5 to S8 and table S2). Although we considered alternative coupled Chron eruption/deposition age and age-depth modeling for data interpretations (table S2) (*36*), they do not affect the conclusions of this study. In addition, the four published CA-ID-TIMS U-Pb dates (*26*) from the Wanhe section (inner shelf; South China) obtained from the Australian National University were used to (i) estimate the boundary ages for comparison with the results of this study and (ii) evaluate the consistency between the datasets from the two laboratories.

## DISCUSSION

### Refined timescale of the Ordovician-Silurian transition

The Hirnantian, which is the last stage of the Ordovician and the time of the LOME (*8*), is characterized by one of the three largest Phanerozoic glaciations (i.e., the Hirnantian glaciation) (*10*, *32*, *37*, *38*). The base of the Hirnantian Stage (i.e., the Katian-Hirnantian boundary) and the top (i.e., Ordovician-Silurian boundary) are estimated to be at 445.2 ± 1.4 and 443.8 ± 1.5 Ma (2σ), respectively, based on the Geologic Time Scale 2020 (*27*). In this study, we obtained three CA-ID-TIMS zircon U-Pb dates from the Wangjiawan North GSSP section that encompass the interval across the Katian-Hir4nantian and Ordovician-Silurian boundaries (Fig. 1). Two ash bed samples (WJW01 and WJW02) were collected 2.4 and 0.85 m below the Ordovician-Silurian boundary, respectively, and one ash bed sample (WJW03) was collected ~0.40 m above the boundary (Fig. 1 and fig. S1). The ages of the three samples are 443.62 ± 0.22, 442.66 ± 0.17, and 442.18 ± 0.32 Ma, respectively. The Bayesian age-depth model based on these dates constrains the ages of the Katian-Hirnantian and Ordovician-Silurian boundaries in the Wangjiawan North section to 442.65 + 0.17/−0.23 and 442.33 + 0.34/−0.33 Ma, respectively.

**Fig. 2.**
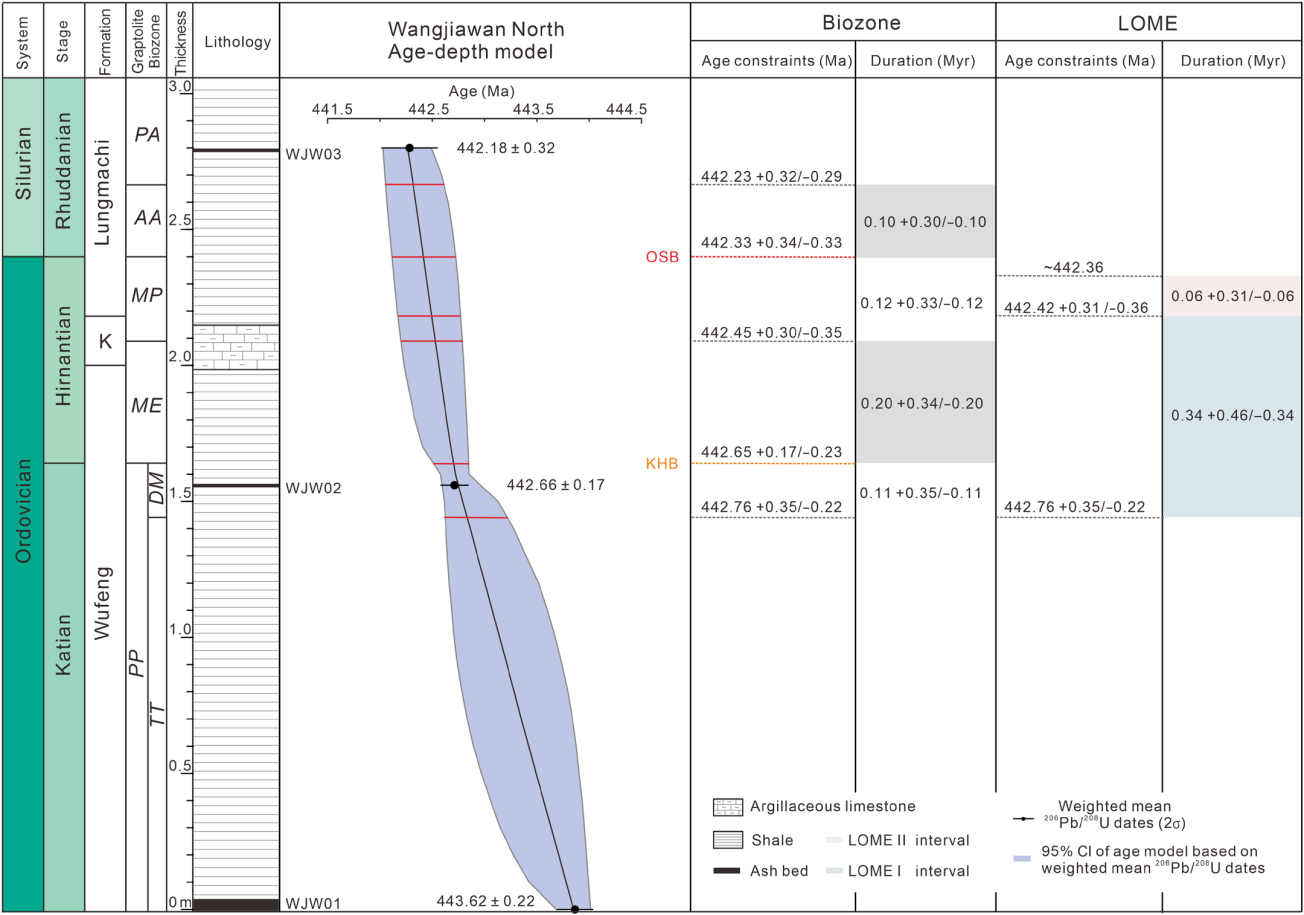
Stratigraphy, zircon ^206^Pb/^238^U dates, Bayesian age-depth model, and interpolated ages of major boundaries for the Wangjiawan North section. The age-depth model is shown with 95% confidence intervals (CIs). OSB, Ordovician-Silurian boundary; KHB, Katian-Hirnantian boundary.

The estimated age of the Katian-Hirnantian boundary in the Wangjiawan North section is consistent with the 442.55 + 0.20/−0.24 Ma age based on the age-depth model for the Shuanghe section (fig. S6). This boundary is estimated to be at 443.21 + 0.37/−0.28 Ma from the age-depth model for the Wanhe section (fig. S8), which appears to be older than that in the Wangjiawan North and Shuanghe sections. The dates from the Beigong section yield an Ordovician-Silurian boundary age of 442.55 + 0.21/−0.28 Ma (fig. S7 and table S2), consistent with the result from the Wangjiawan North section. Therefore, we suggest that the two interpolated dates from the Wangjiawan North section represent the ages of the bases of the Hirnantian Stage and Silurian System, respectively, leading to a duration of 0.32 + 0.38/−0.32 Myr for the Hirnantian Stage (Fig. 2). The updated ages for the two chronostratigraphic boundaries, along with the age estimates for the other biozone boundaries (table S2) from the age-depth models, provide a refined timescale for the Ordovician-Silurian transition.

### Timing and tempo of the LOME

The LOME has generally been divided into two phases (I and II). LOME I began in the *D. mirus* subzone (*3*), which is ~0.19 m below the base of the Hirnantian Stage in the Wangjiawan North section. The depositional age of this horizon is estimated to be 442.76 + 0.35/−0.22 Ma from the age-depth model (Fig. 2). The precise onset of LOME I in the Shuanghe section cannot be determined because of the absence of the *D. mirus* subzone, but it must be earlier than 442.55 + 0.20/−0.23 Ma (fig. S6). The age-depth model for the Wanhe section constrains the onset of LOME I to 443.44 + 0.36/−0.37 Ma (fig. S8). The age from the Wanhe section appears to be older than that from the Wangjiawan North section, and the estimated ages of the Katian-Hirnantian boundary from the two sections show a similar difference. The difference in the estimated onset of LOME I between the two sections is 0.68 + 0.50/−0.43 Myr, and the difference between the estimated Katian-Hirnantian boundary ages is 0.56 + 0.41/−0.36 Myr. The results from the Wanhe section are systematically older than those from the Wangjiawan North section (Fig. 1 and table S2). Given that these zircon U-Pb dates from the two sections were obtained from two laboratories using different U-Pb tracers, we reconstructed age-depth models for the Wangjiawan North and Wanhe sections using dates with Y uncertainties. However, the aforementioned differences remain resolvable (fig. S9). We speculate that this discrepancy could be caused by the coarse resolution of the graptolite biostratigraphy in the Wanhe section (*26*, *27*), the diachronous onset of LOME I and biozones in different depositional environments (*3*, *28*), or interlaboratory bias.

LOME II began in the lower part of the *M. persculptus* biozone, which is ~0.21 m below the Ordovician-Silurian boundary in the Wangjiawan North section (the top of the Kuanyinchian Bed) (*28*, *31*,*32*, *39*). The age-depth model for the Wangjiawan North section constrains the depositional age of this horizon to 442.42 + 0.31/−0.36 Ma. The Shuanghe section is less condensed than the Wangjiawan North section, and it has been well calibrated using graptolite biozones (*40*) and organic C isotopic data (Fig. 1). Therefore, the three zircon U-Pb dates from the Shuanghe section, along with the correlated Ordovician-Silurian boundary age (discussed above), were used to test the age estimate from the Wangjiawan North section. The age-depth model for the Shuanghe section (fig. S6) yields an onset age for LOME II of 442.37 + 0.26/−0.27 Ma, which is consistent (i.e., within uncertainties) with the result from the Wangjiawan North section. We note that the ash bed (AJB02) dated at 442.63 ± 0.21 Ma in the Beigong section is close to the horizon marking the onset of LOME II. This date is within uncertainties of the estimate from the Wangjiawan North section. Given that the Beigong section is only partially exposed and the biostratigraphy and chemostratigraphy of this section are of low resolution or lacking, the estimated age of 442.42 + 0.31/−0.36 Ma from the Wangjiawan North section is suggested to represent the onset of LOME II.

**Fig. 3.**
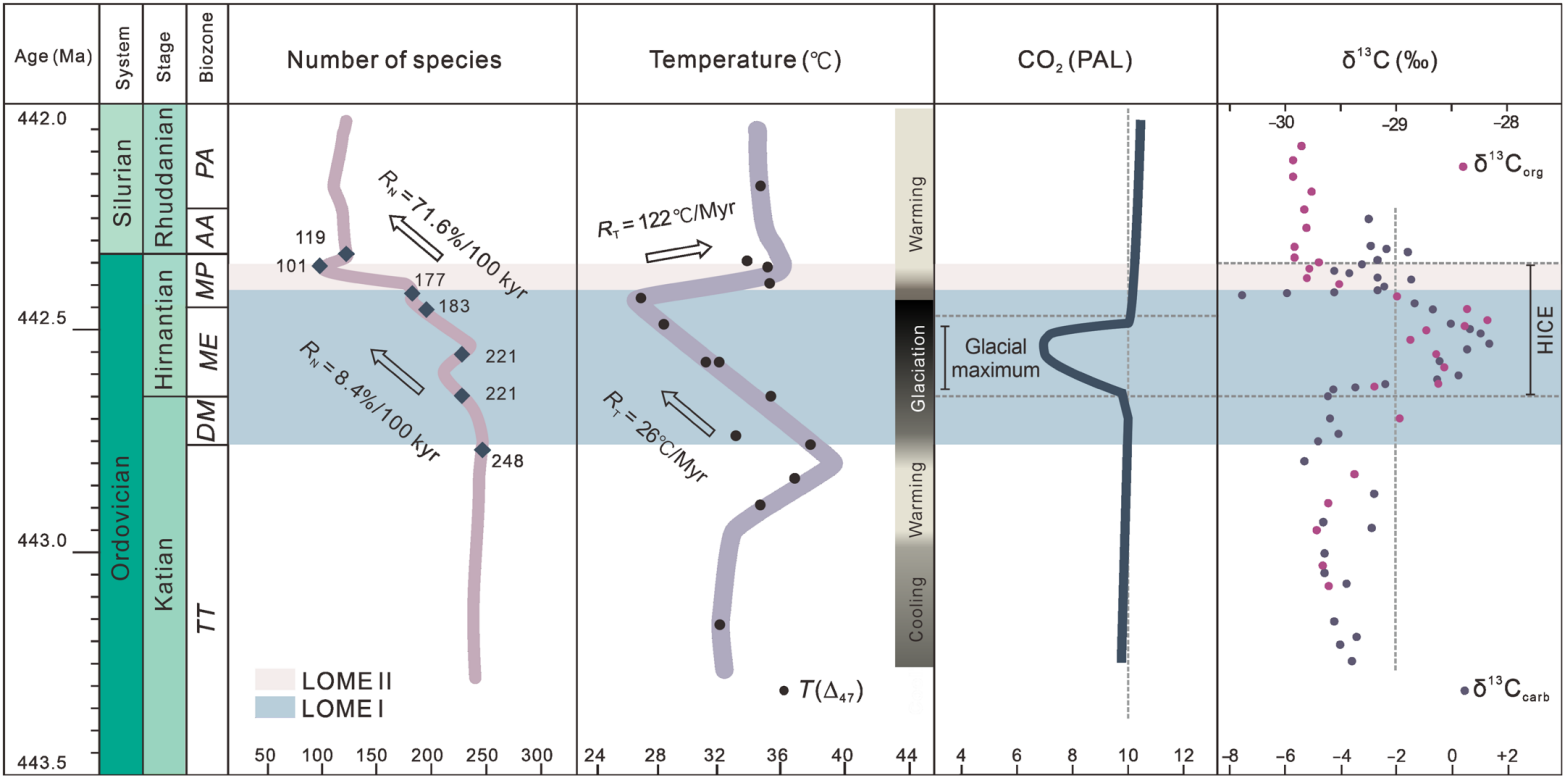
Refined timescale of the Ordovician-Silurian transition with the age-calibrated biodiversity, temperature, atmospheric CO_2_, and C isotope changes. The species diversity curve is modified from (*6*). The paleotemperature curve is modified from (*9*). The curve of the atmospheric CO_2_ levels relative to present-day is from (*72*). The δ^13^C profile is from (*35*, *73*). HICE, Hirnantian carbon isotope excursion.

LOME II ended just below the Ordovician-Silurian boundary and is often marked by the appearance of the Edgewood-Cathay Fauna (*41*, *42*). A biodiversity curve calibrated to our chronostratigraphic data indicates that biodiversity reaches a minimum at ~442.36 Ma (Fig. 3) and, therefore, we regard this date to mark the end of LOME II. The calibrated ages for the onset of LOME I, onset of LOME II, and end of LOME II enable us to calculate the durations and rates of the two extinction phases. The actual extinction rates of the two phases were probably nonlinear, but given the resolution of the presented and interpolated dates, the mean extinction rates for the two phases were calculated in this study. The LOME I began at 442.76 + 0.35/−0.22 Ma and ended at 442.42 + 0.31/−0.36 Ma, with a duration of 0.34 + 0.46 /−0.34 Myr. Applying these age constraints to the available biodiversity data (*6*) reveals that the species abundance declined at a mean rate of 8.4% per 100 thousand years (kyr), resulting in a ~28.6% loss in species richness during LOME I. The LOME II began at 442.42 + 0. 31/−0.36 Ma and ended at 442.36 Ma, with a duration of 0.06 + 0.31/−0.06 Myr. The species abundance declined at a mean rate of 71.6% per 100 kyr during LOME II, which is about eight times faster than that during LOME I, and resulted in a ~42.9% loss of species richness (Fig. 3). The distinct extinction rates obtained from the age-calibrated Late Ordovician biodiversity curve, along with the differences in the species that became extinct, provide robust evidence that the LOME occurred in two phases. The entire LOME had a duration of 0.40 + 0.46/−0.34 Myr, which is much shorter than the previous estimates of 2.2 Myr from constrained optimization (CONOP) analysis (*6*) and 0.98 Myr from cyclostratigraphic constraints (*25*).

### Rate of climate change controlled the tempo of the LOME

**Fig. 4.**
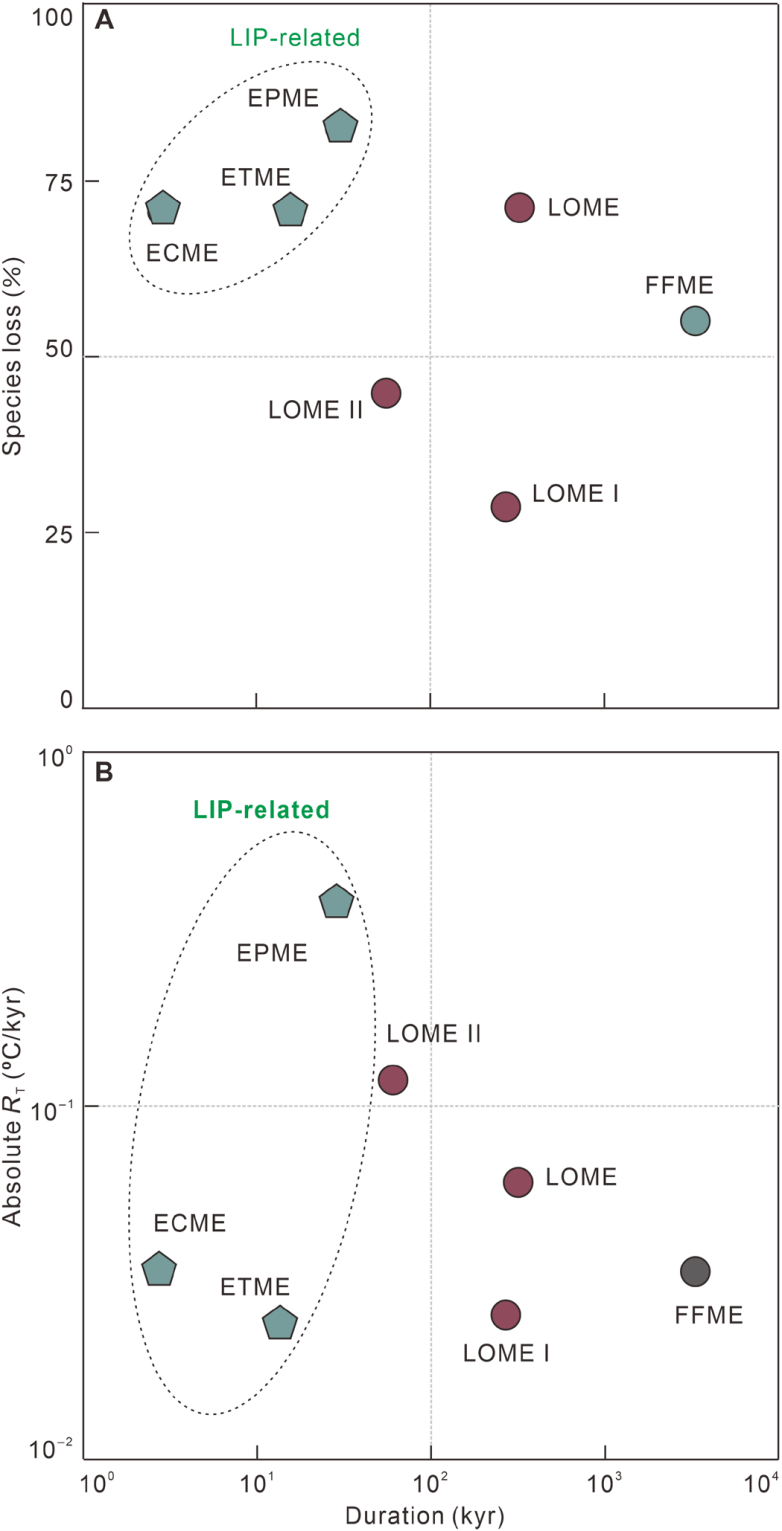
Extinction magnitudes of the Phanerozoic Big Five mass extinction events and their associated temperature changes. The species loss (**A**) and mean rates of temperature change (**B**) are plotted against the durations of the Big Five mass extinction events.

Although the total duration of the LOME obtained in this study is much shorter than previous estimates, it ranks as the second longest in terms of duration of the Big Five mass extinction events and is only slightly shorter than the Frasnian-Famennian mass extinction (Fig. 4) (*43*). In contrast, the mass extinctions that occurred at the end-Permian (EPME), end-Triassic (ETME), and end-Cretaceous (ECME) had much shorter durations (Fig. 4) (*36*, *44*, *45*). Although there are various hypotheses for the causes of extinction events, the EPME, ETME, and ECME are all suggested to have been associated with large igneous provinces (LIPs) or extraterrestrial impacts (*36*, *45*–*49*). These near-instantaneous catastrophic events had profound effects on the biosphere and are thought to have caused abrupt decreases in biodiversity and severe ecological crises (Fig. 4) (*36*, *45*, *50*, *51*).

Possible Ordovician LIPs were emplaced in southeastern Siberia and northern Iran (*52*–*54*). The Suordakh dike swarm in southeastern Siberia has been dated to 454 ± 10 Ma, and its areal extent is estimated to be 35,000 to 40,000 km^2^ (*53*). The large uncertainty on the emplacement age and its relatively small area make a causal linkage between the Suordakh dike swarm and the LOME unlikely. Several phases of volcanism occurred during the Middle-Late Ordovician in northern Iran (*55*). These are regarded as being part of a potential LIP named the Alborz LIP (*55*). Phase 4 of this volcanism has been dated to 442.5 ± 2.5 Ma, which is probably coincident with the LOME within uncertainties (*55*). The areal extent of the possible Alborz LIP is estimated to be 30,000 km^2^, and the extent of its phase 4 volcanism must be substantially smaller than this estimate. Hence, the areal extent of the phase 4 volcanism is at least an order of magnitude smaller than the LIPs hypothesized to have caused the EPME, ETME, and ECME, and its effects on the LOME are yet to be established.

Conodont O isotopes and carbonate clumped isotopes (∆_47_) both indicate that notable temperature changes occurred during the LOME (*9*, *56*, *57*). Sea surface temperatures plummeted by 9°C (from 37.5° to 28.5°C) from the late Katian to early-middle Hirnantian (*9*), coinciding with the Hirnantian glacial maximum, a global sea level low stand, and LOME I. The timescale of LOME I constrained by this study indicates that the mean rate of temperature decrease during LOME I was 26°C/Myr (Fig. 3). In the late Hirnantian, the glaciation ceased and global surface temperatures increased from 28.5° to 35.8°C, resulting in a mean rate of temperature increase of 122°C/Myr during LOME II (Fig. 3). Overall, the absolute temperature change during the LOME was ~16.3°C, and the mean rate of temperature change was ~41°C/Myr. The absolute temperature change and its mean rate of change during the LOME are above the proposed thresholds of temperature change for mass extinctions, which are >5.2°C and > 10°C/Myr (*21*), respectively, implying that rapid climate change may have been the main cause of the LOME.

**Fig. 5.**
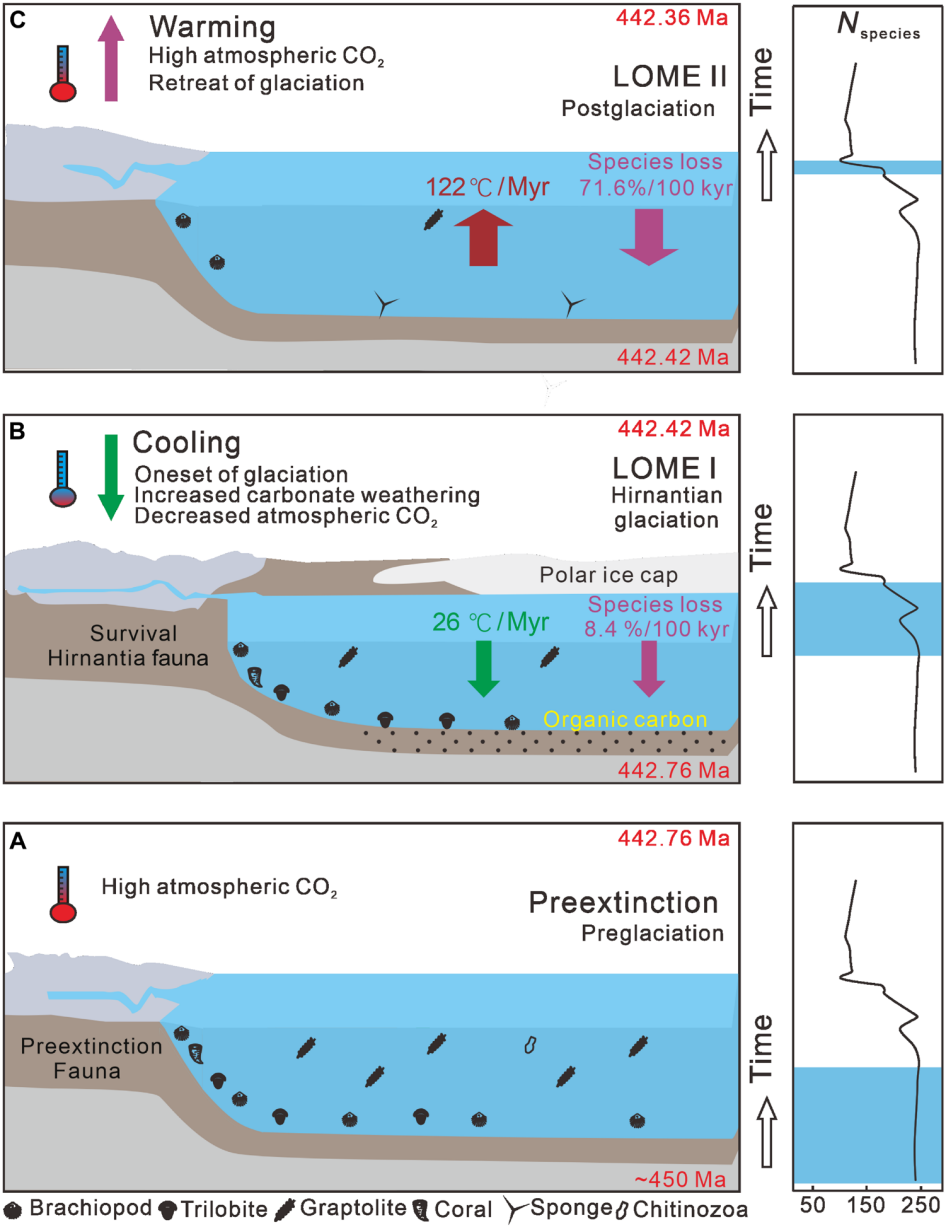
Schematic diagram of species loss controlled by climate change during the LOME. The biodiversity curve is modified from (*6*), and the faunal distribution is modified from (*29*, *34*) [(*34*), https://creativecommons.org/licenses/by/4.0/]. (**A**) The late Ordovician Earth-Life system prior to the LOME was characterized by high biodiversity, high atmospheric CO_2_, and high seawater surface temperature. (**B**) The LOME I, with a mean biodiversity loss rate of 8.4% per 100 kyr, was associated with a mean temperature decrease of 26°C per myr and the Hirnantian glaciation. (**C**) The LOME II which had a higher mean biodiversity loss rate of 71.6% per 100 kyr, coincided with a rapid temperature increase of 122°C per myr and the retreat of Hirnantian glaciation.

In addition, the mean extinction rates of the two phases of the LOME are positively correlated with the mean rates of temperature change during their corresponding time intervals (Fig. 5). In detail, the mean rate of extinction during LOME II was about eight times higher than that during LOME I, while the mean rate of temperature change during LOME II was about four times higher than that of LOME I (Fig. 3). The mean rate of extinction of surviving species from the first phase in response to rapid climatic warming was higher than that of the Late Ordovician warm-water fauna in response to climate cooling (Fig. 3). Modeling suggests that extinction proceeds differently under greenhouse and icehouse conditions and that high low-latitude extinction can be partly counterbalanced by the development of refugia at higher latitudes during global warming, resulting in relative resilience to warming-induced extinctions in cool environments (*58*, *59*). The mean extinction rate of the cold-water Hirnantian fauna, along with many other open marine fauna, in response to climatic warming appears to have been higher than predicted by the modeling, which may have been due to the relatively high global surface temperatures even during the Hirnantian glaciation (*9*, *56*) or the rapid climate change that ecosystems could not adapt to (Fig. 5). Hence, the rate of climate change, including that of the mean temperature and the rapid switch between greenhouse and icehouse conditions, may have been a primary control on the tempo of the LOME.

## MATERIALS AND METHODS

### U-Pb geochronology

The samples used for U-Pb geochronology weighed about 5 kg each. They were thoroughly dried and then mechanically crushed to a particle size of 60 to 80 mesh. The rock powder was then subjected to conventional density and magnetic separation techniques. Subsequently, prismatic or needle-like zircon grains were selected under a binocular microscope, and reflected light photographs were taken at the British Geological Survey, Nottingham, UK (fig. S4).

These grains were dated using the CA-ID-TIMS U-Pb method at the British Geological Survey. The grains were pretreated using a CA technique, which involved thermal annealing in a furnace at 900°C for 60 hours in quartz beakers, followed by partial dissolution in 29 M HF at 180° to 195°C in high-pressure vessels for 12 hours. The chemically abraded grains were rinsed three or four times with several hundred microliters of 4 M HNO_3_ and 6 M HCl to remove the leachates before spiking with the mixed EARTHTIME ET2535 tracer (*60*, *61*). The single zircons were dissolved in ~120 μl of 29 M HF with a trace amount of 4 M HNO_3_ at 220°C for 60 hours, and, subsequently, Pb and U were purified by HCl-based anion exchange resin column chemistry.

Pb and U were loaded together onto single outgassed Re filaments along with a silica gel emitter solution. The isotopic ratios of Pb and U were measured with a Thermo Fisher Scientific Triton instrument equipped with an ion-counting system. Lead isotopes were measured in dynamic mode on a MassCom secondary electron multiplier and corrected for mass bias in real time based on the measured ^202^Pb/^205^Pb ratios. Uranium isotopes were measured as dioxide ions (UO_2_^+^) either in static mode on Faraday collectors equipped with 10^12^-ohm resistors for intensities greater than 4 mV, or in dynamic mode for lower intensities. Uranium mass fractionation was corrected in real time on the basis of the isotopic composition of the ET2535 tracer. An oxide correction based on an independently determined ^18^O/^16^O ratio of 0.00205 ± 0.00004 was applied to the measured U isotopic ratios. The sample ^238^U/^235^U ratio was assumed to be 137.818 ± 0.045 (*62*). Initial Th/U disequilibrium was corrected using radiogenic ^208^Pb and a magmatic Th/U ratio of 2.8 ± 0.5 (1σ). All common Pb was assumed to be from laboratory blank. The blank Pb isotopic composition is ^206^Pb/^204^Pb = 18.10 ± 0.27, ^207^Pb/^204^Pb = 15.55 ± 0.14, and ^208^Pb/^204^Pb = 37.82 ± 0.41 (1σ).

### Age interpretation and Bayesian age-depth model

Seventy-two single-zircon U-Pb dates were obtained from 10 tuff samples using the CA-ID-TIMS technique. Tripoli and ET_Redux algorithms were used for data reduction, age calculations, and propagation of uncertainties (*63*–*65*). The isotopic data are listed in table S3 and shown in figs. S5 to S8. The data are reported following the recommended format of Condon *et al.* (*66*). All samples were used to estimate the depositional ages with the weighted-mean ^206^Pb/^238^U model (table S2). Uncertainties are expressed at the 2σ level as ±*X*/*Y*/*Z*, where *X* represents the analytical uncertainty, *Y* incorporates the tracer uncertainty (*67*), and *Z* accounts for the combined uncertainties from the analysis, tracer, and decay constants (*68*). The zircon U contents are variable, with relatively low U levels in the zircons from sample WJW03, resulting in only a few picograms of radiogenic Pb. Consequently, the age uncertainties for this sample are relatively high.

We used the Chron.jl package (*36*) (https://github.com/brenhinkeller/Chron.jl) to construct age-depth models for all sections, enabling the estimation of the ages of key chronostratigraphic boundaries and the duration of the LOME (Fig. 2 and figs. S5 to S8). The age-depth model used the measured U-Pb dates and associated uncertainties, stratigraphic positions, and Bayesian statistical interpolation with an MCMC algorithm to calculate numerical ages throughout the entire section with 95% confidence intervals. The MCMC algorithm was also used to estimate the uncertainties of the durations of the biozones and LOME.

### Organic carbon isotopes

All samples were crushed to powder (>200 mesh) for δ^13^C_org_ analysis. The sample powders were first leached seven times in 15 vol % HCl and then boiled in 15 vol % HCl for 1 hour to remove any carbonate components. The residue was then leached/washed in deionized water until the pH reached 7 and dried at 60°C overnight. The isotopic analyses were conducted with a Flash 2000 elemental analyzer connected to a Thermo Fisher Scientific DELTA V isotope ratio mass spectrometer. The isotope data are reported in per mil (‰) notation relative to the Vienne Pee Dee Belemnite standard. The analyses were conducted at the Technical Services Centre, Nanjing Institute of Geology and Palaeontology, Chinese Academy of Sciences, Nanjing, China.

### The magnitude and mean rate of biodiversity loss.

The magnitude ( ΔN ) of biodiversity loss during the mass extinction event was calculated as follows

$$\Delta N=N_{0}–N_{1}$$ (1)

where $N_{0}$ and $N_{1}$ represent the initial and end species richness of the mass extinction event, respectively.

The timescale Δt represents the duration of the mass extinction event, calculated as follows

$$\Delta t=t_{0}–t_{1}$$ (2)

The mean rate ( $R_{N}$ ) of the relative change in the biodiversity loss was calculated as follows

$$R_{N}=\Delta N/\left(N_{0}\times \Delta t\right)\times 100\%$$ (3)

### Magnitude and mean rate of temperature change

The maximum magnitude ( ΔT ) of temperature change during the mass extinction event was calculated as follows

$$\Delta T=T_{1}-T_{0}$$ (4)

where $T_{0}$ and $\;T_{1}$ represent the initial and end temperatures of a warming/cooling event, respectively.

The rate ( $R_{T}$ ) of temperature change was calculated as follows

$$R_{T}=\text{absolute}\;\left(\Delta T\right)/\Delta t$$ (5)

The ratio R represents the mean rate of temperature change in a single warming/cooling event during the mass extinction event.

## Supplementary Materials

### The PDF file includes:

Supplementary Text Figs. S1 to S9 Legends for tables S1 to S3 References
