# Supplementary material for: Tempo of the Late Ordovician mass extinction controlled by the rate of climate change
Source: Sci Adv. 2025 May 30;11(22):eadv6788. doi: 10.1126/sciadv.adv6788 (PMC12124363; doi:10.1126/sciadv.adv6788)
Supplement: Supplementary file 1 — Supplementary Text Figs. S1 to S9 Legends for tables S1 to S3 References [file sciadv.adv6788_sm.pdf]

Science Advances
AAAS

Supplementary Materials for

Tempo of the Late Ordovician mass extinction controlled by the rate of climate change

Zhutong Zhang *et al.*

Corresponding author: Chuan Yang, cyang@nigpas.ac.cn; Xian-Hua Li, lixh@gig.ac.cn

Sci. Adv. **11**, eadv6788 (2025)
DOI: 10.1126/sciadv.adv6788

The PDF file includes:

Supplementary Text
Figs. S1 to S9
Legends for tables S1 to S3
References

Other Supplementary Material for this manuscript includes the following:

Tables S1 to S3

Supplementary Text

S1 Geological background

The basic framework of the Chinese topography was formed during the Mesozoic Yanshan Orogeny (*74*). The South China Palaeoplate can be divided into three different types of tectonic-palaeographic regions, including Yangtze Platform, Jiangnan Slope, Zhujiang Basin, with discontinuous clusters of emergent landmasses or arches (oldlands) (*75-77*) (Fig. 1B). During the Late Ordovician, South China Palaeoplate evolved from being part of the Gondwana supercontinents to independent and stable tectonic unit (*78-80*) and straddled the equator (*81*). At the end of the Late Ordovician (Hirnantian stage), a sudden glaciation in Gondwana caused a rapid drop in sea level, leading to the formation of glacial deposits in high-latitude regions and shallow water deposits in low-latitude areas. Estimates of sea-level change vary significantly, ranging from 50-100 meters in Oslo, Norway (*82*) to 30-60 meters on Anticosti Island, Quebec, Canada (*83, 84*), where mass flow deposits or turbidites may have disrupted deep shelf sedimentation. In contrast, sea-level change on the Yangtze Shelf Sea in South China was likely less than 20 meters and was marked only by the appearance of Hirnantian fauna in the Kuanyinchiao Bed (*39, 85*). Additionally, graptolite biozones are well-defined in South China and show strong correlation with those in other regions globally (*35, 86*). Much of the South China palaeoplate was covered by a restricted epicontinental sea and received organic-rich mud deposits that became the black shale sequence of the Wufeng Formation. At some near-shore localities in the southwestern part of the Yangtze Platform, argillaceous limestone layers developed with intercalated graptolitic black shales, forming the Daduhe Formation of the Wanhe section. Four sections, representing a range from proximal to distal areas on the Yangtze Shelf Sea, were selected for this study. Samples were collected from one middle shelf (Wangjiawan North), a mid-outer shelf (Shuanghe) and an outer shelf to slope section (Beigong), primarily spanning the Late Ordovician (Katian Stage) through to the early Silurian (Rhudanian stage). Additionally, we revised the zircon data from ash layers in the Wanhe section (inner shelf), which was initially reported by the ref. (*26*).

S2 Stratigraphy of the studied sections and geochronologic sampling

The Wangjiawan North section (30º58′56″N, 111°25′21″E) is located in Yichang, Hubei Province, which possesses continuity of sedimentation and biozonation with completeness of exposures, abundant and well-preserved graptolites and shelly fossil spanning from Late Ordovician to early Silurian. The strata across the Ordovician-Silurian boundary at Wangjiawan North include the Wufeng Formation, Kuanyinchiao Bed, and Lungmachi Formation in ascending order (Fig. 1). The biozonation through these lithostratigraphic units was defined by the ref. (*35, 87*). A stratigraphic level 0.39 meters below the base of the Kuanyinchiao Bed in the Wangjiawan North section be designated as the Global Stratotype Section and Point (GSSP) for the Hirnantian Stage (*35*). The following sequence of events can be distinguished in this section(Fig 1): (i) a 3‰ positive $\delta^{13}C$ isotopic excurison over *Metabolograptus extraordinarius* Biozone; (ii) the main extinction event between *Diceratograptus mirus* and *Metabolograptus persculptus*; and (iii) the base of the Hirnantian Stage is coincident with the First Appearance Datum (FAD) of the graptolite *Metabolograptus extraordinarius* and the Ordovician-Silurian boundary can be defined by the FAD of the graptolite *Akidograptus ascensus* in this section. At this level, two ash bed samples (WJW01 and WJW02) were collected 2.0 meters and 0.45 meters below Kuanyinchiao bed in the Wangjiawan North section respectively. Further up section at Wangjiawan North, one ash bed

(WJW03) was collected from the Silurian strata (the bottom of the Lungmachi Formation), approximately 0.67 meters above the Kuanyinchiao bed (Figs 1 and S1).

The Shuanghe section (28º23′52.9″N, 104°52′26.7″E) is located in Changning County, Sichuan Province, which is well calibrated biostratigraphically based on the literature (*37*). The lithology of this section is consistent with that of the Wangjiawan North section. The Wufeng and Lungmachi Formations are primarily composed of black calcareous shale with minor black mudstone. The Wufeng Formation includes abundant graptolites and has a thickness of approximately 10 meters. The Kuanyinchiao Bed mainly consists of grey shelly mudstone, with a total thickness of about 60 centimeters. Three ash bed samples (SHW01, 03, 05) and 68 inorganic carbon isotope samples have been collected in this section. Sample SHW05 was collected from a 4 cm-thick yellowish-gray ash bed near the base of the Hirnantian Stage (Figs 1 and S2).

In Beigong section of Anhui Province, which is a continuous and fossiliferous OSB section with well-exposed outcrop and interpreted palaeogeographically to be part of the Lower Yangtze Platform, characterized by graptolitic shale facies. A recently reported deep-water siliceous sponge assemblage from this section also suggests that the Beigong area developed an open marine ecosystem along the platform margin, characterized by relatively deep-water, low-energy, and oxygen-deficient conditions during the Late Ordovician to early Silurian period (*39*). Above the Wufeng Formation lie the black calcareous mudstones of the Xinkailing Bed (corresponding to the Kuanyinqiao Bed of Upper Yangtze Platform), which contain graptolites, trilobites, brachiopods, nautiloids, and other shelly fossils that suggest a mixed facies and represent the upper part of the *M. extraordinarius* Biozone. The Kaochiapien Formation (in the Upper Yangtze Platform the equivalent Formation is defined as the Lungmachi Formation) conformably overlies the Xinkailing Bed that contains black siliceous and calcareous mudstone. The bottom of the Kaochiapien Formation is approximately correlated with the OSB. According to the graptolites from the Wufeng and Kaochiapien formations, three graptolite biozones could be recognized in ascending order: *Metabolograptus extraordinarius* biozone, *Metabolograptus persculptus* biozone, and *Akidograptus ascensus* biozone. The index fossils *M. persculptus* and *A. ascensus* are not found here, but other important species such as *Avitograptus avtitus*, *Normalograptus laciniosus*, *N.normalis*, *Neodiplograptus shanchongensis*, and *Ne. modestus* confirm the presence of the *M. persculptus* Biozone and *A. ascensus* biozone at Beigong section (*88*). In the Xinkailing bed, the brachiopod species *Aegiromenella planissima* (Reed), consisting of small individuals (3-5 mm), and *Paromalomena polonia* (Temple) were identified, belonging to the *Paromalomena-Aegiromenella* assemblage (*89*) and attributed to the *Hirnantia* fauna. Additionally, trilobites such as *Mucronaspis (Songxites) wuningensis* Lin, *Eoleonaspis sinensis (35)* Sheng, and *Platycoryphe* sp., along with gastropod, cephalopod, and other shell fossils, were also observed. A total of 4 samples of ash beds (AJB01-04) were collected from this section for U-Pb geochronological analysis. Sample AJB02 was collected from the top of the Xinkailing Bed. (Figs 1 and S3). Samples AJB03 and AJB04 were collected from 0.45 m below the OSB and 1.15 m above the OSB, respectively (Figs 1 and S3).

The Wanhe section is situated in Yongshan County, northeastern Yunnan Province, southwestern China (27°45′18″N, 103°27′58″E). The lithostratigraphy and biostratigraphy of this section indicate a continuous sedimentary succession, which has been thoroughly described by the literature (*90*). The Daduhe Formation, measuring 24.3 meters in thickness, is composed of thin- to medium-bedded argillaceous limestone and calcareous shale, interspersed with numerous K-

bentonite layers ranging from 1-2 mm to several centimeters in thickness. The Kuanyinchiao Bed, 50 cm thick, contains the characteristic *Hirnantia* brachiopod fauna. Five successive graptolite biozones, spanning from the upper Katian to the lower Hirnantian, have been identified in the Daduhe Formation. These biozones correlate well with those found in the widespread Wufeng Formation on the Yangtze Platform (*26, 90*). Four dated ash beds in this section are only from Ordovician, the sample AGM120 comes from 1.85 meters below the OSB, approximately 1 meter above the base of the Hirnantian stage (Fig. 1).

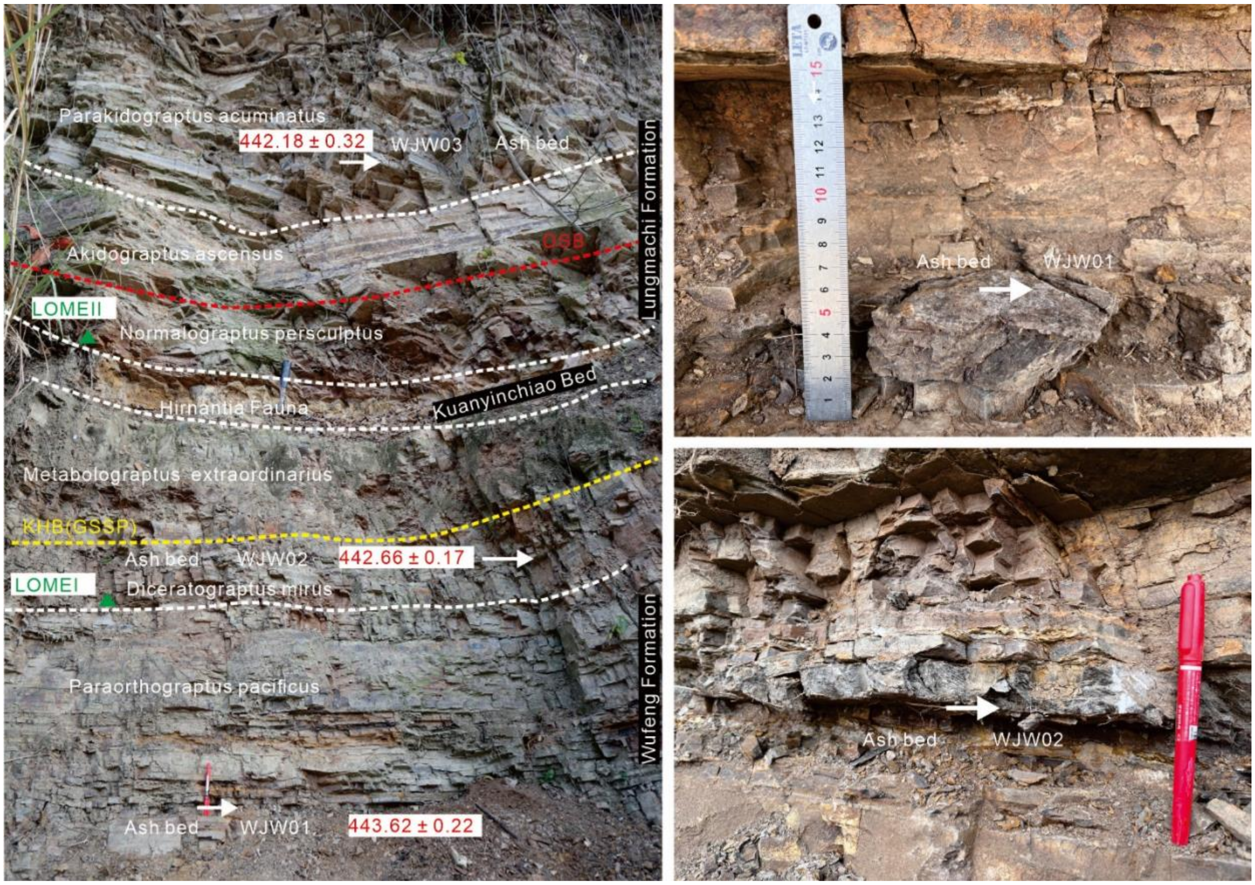

fig. S1. Outcrop photos of the Wangjiawan North section. The subdivision of graptolite biozone is based on the ref. (*35*). (Photo Credit: Zhutong Zhang，Institute of Geology and Geophysics, Chinese Academy of Sciences)

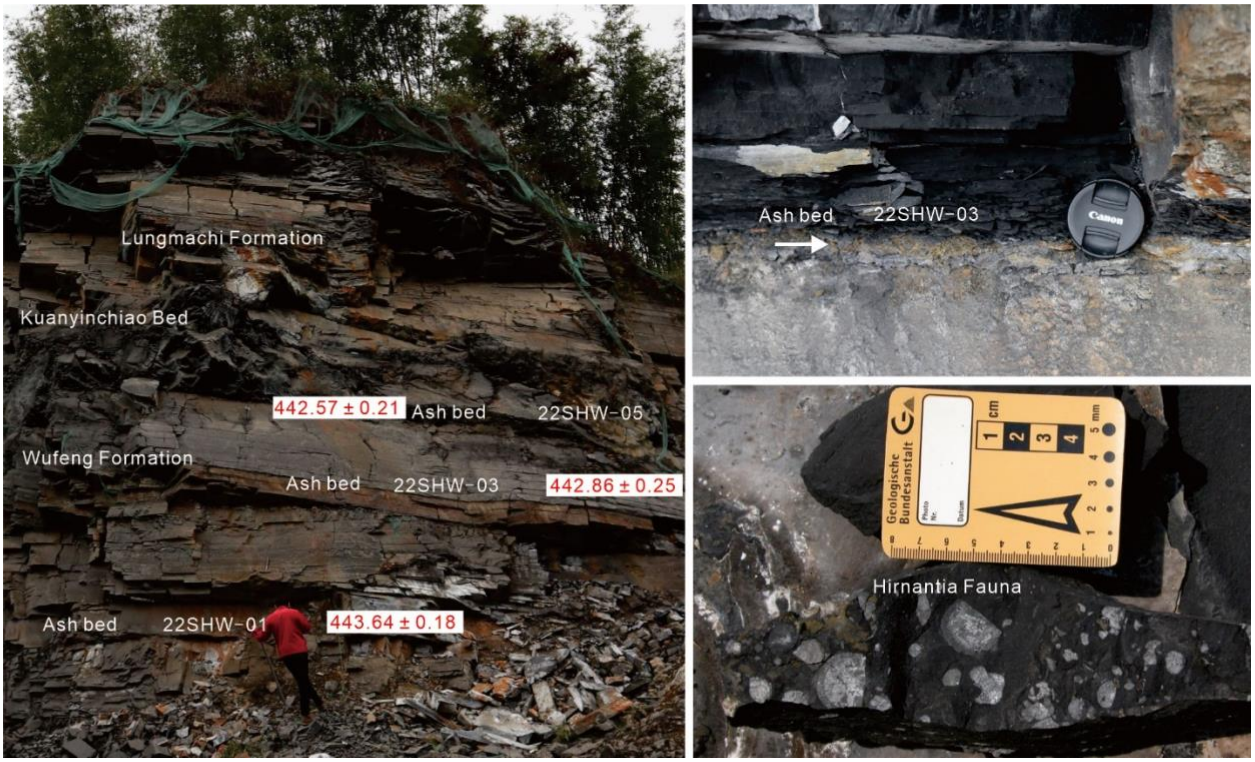

fig. S2. Outcrop photos of the Shuanghe section. (Photo Credit: Zhutong Zhang，Institute of Geology and Geophysics, Chinese Academy of Sciences)

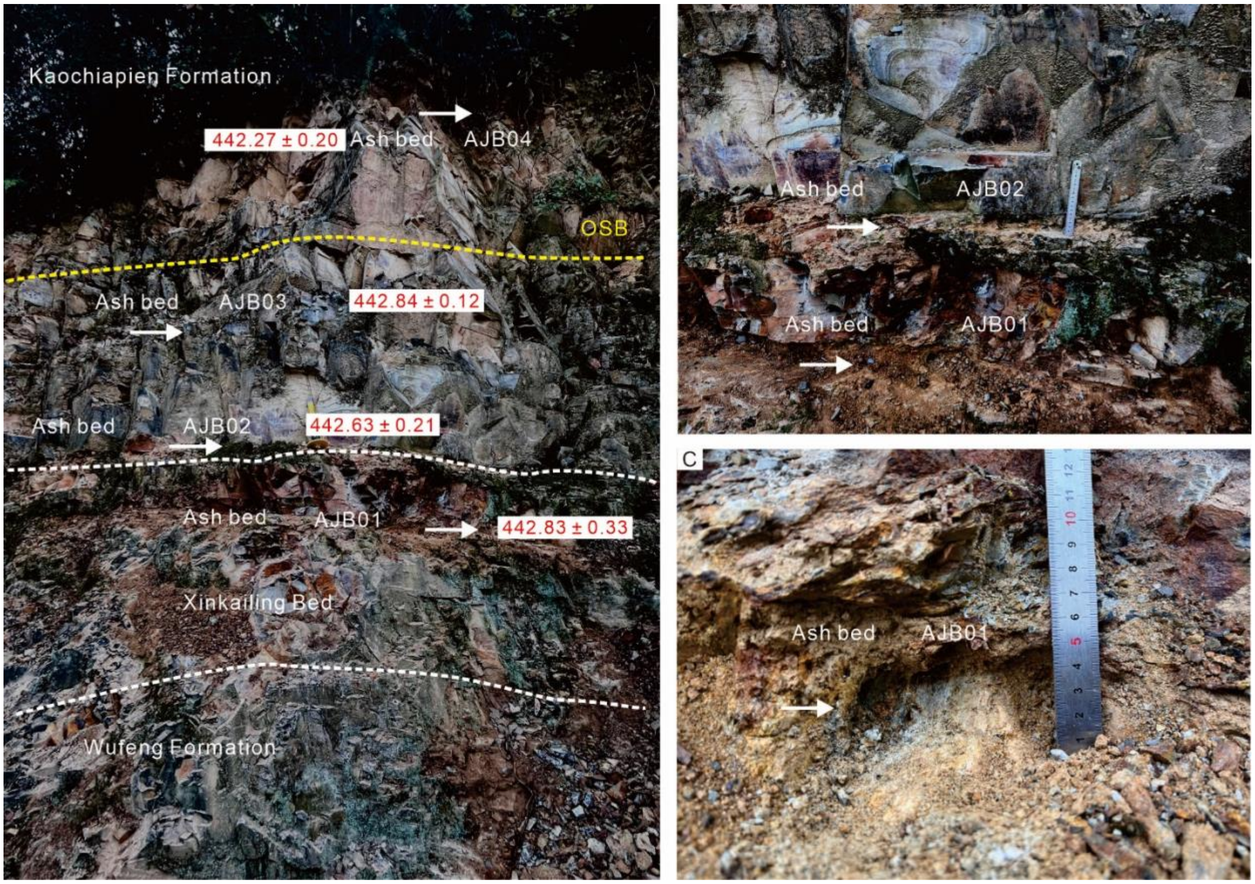

fig. S3. Outcrop photos of the Beigong section. (Photo Credit: Zhutong Zhang，Institute of Geology and Geophysics, Chinese Academy of Sciences)

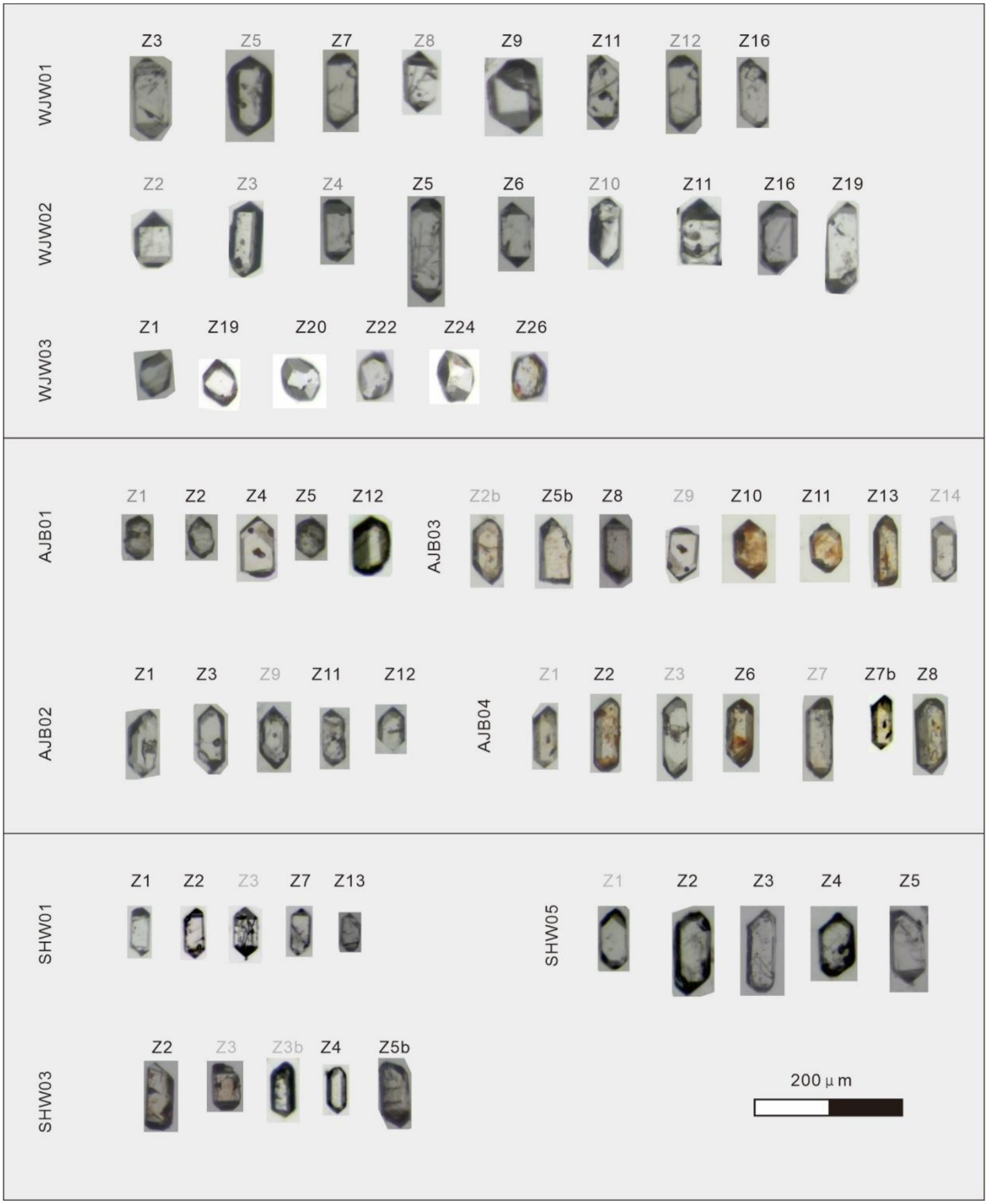

fig. S4. Optical images of zircon grains under reflected light.

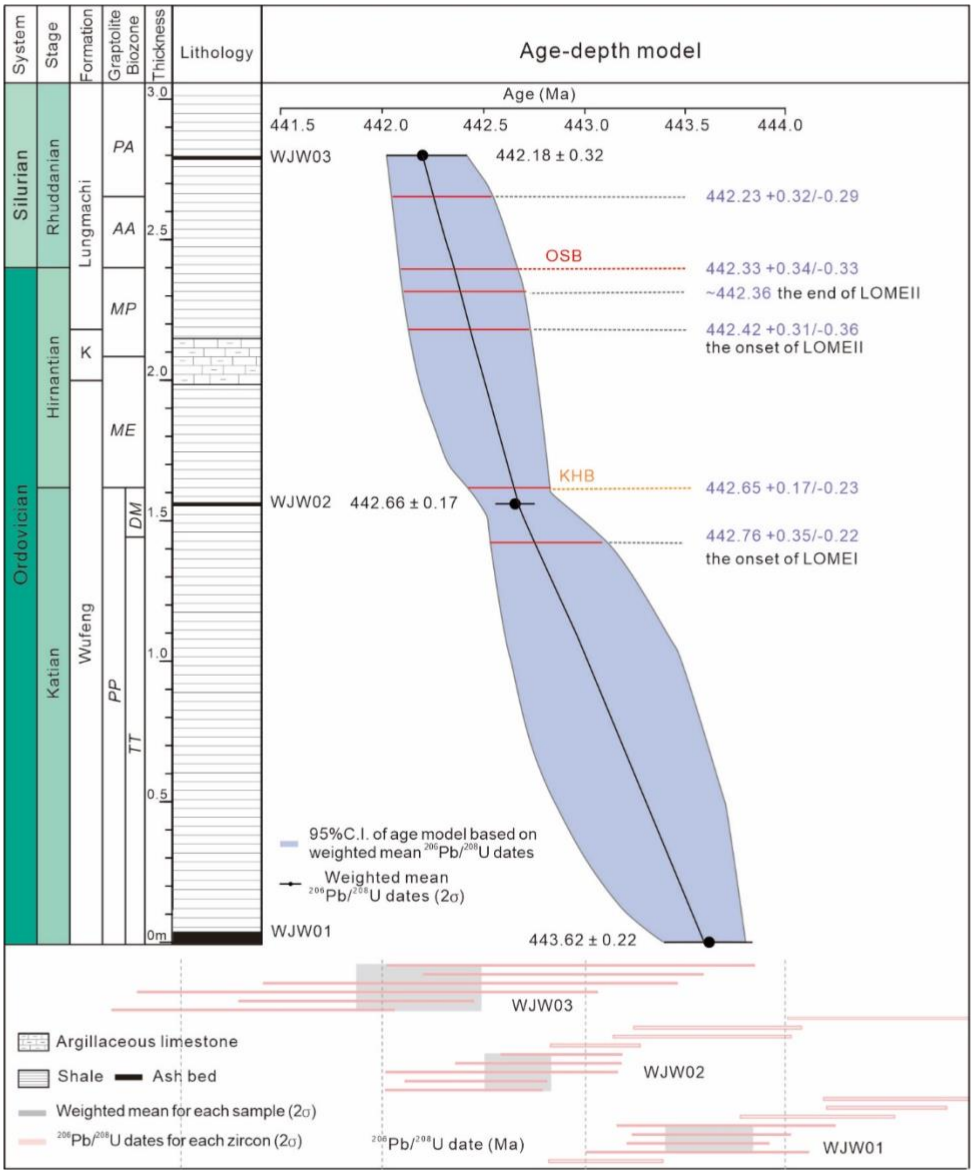

fig. S5. Stratigraphy, geochronology and Bayesian age-depth model for the Wangjiawan North section. Estimated ash bed depositional ages based on weighted mean $^{206}Pb/^{238}U$ dates (in black) and their stratigraphy positions were input to the Markov Chain Monte Carlo (MCMC) algorithm of the Chron package (*36*) to construct a Bayesian age-depth model with 95% confidence interval (shading). The biostratigraphic calibration is based on ref (*35*). The purple represents the interpolated depositional ages.

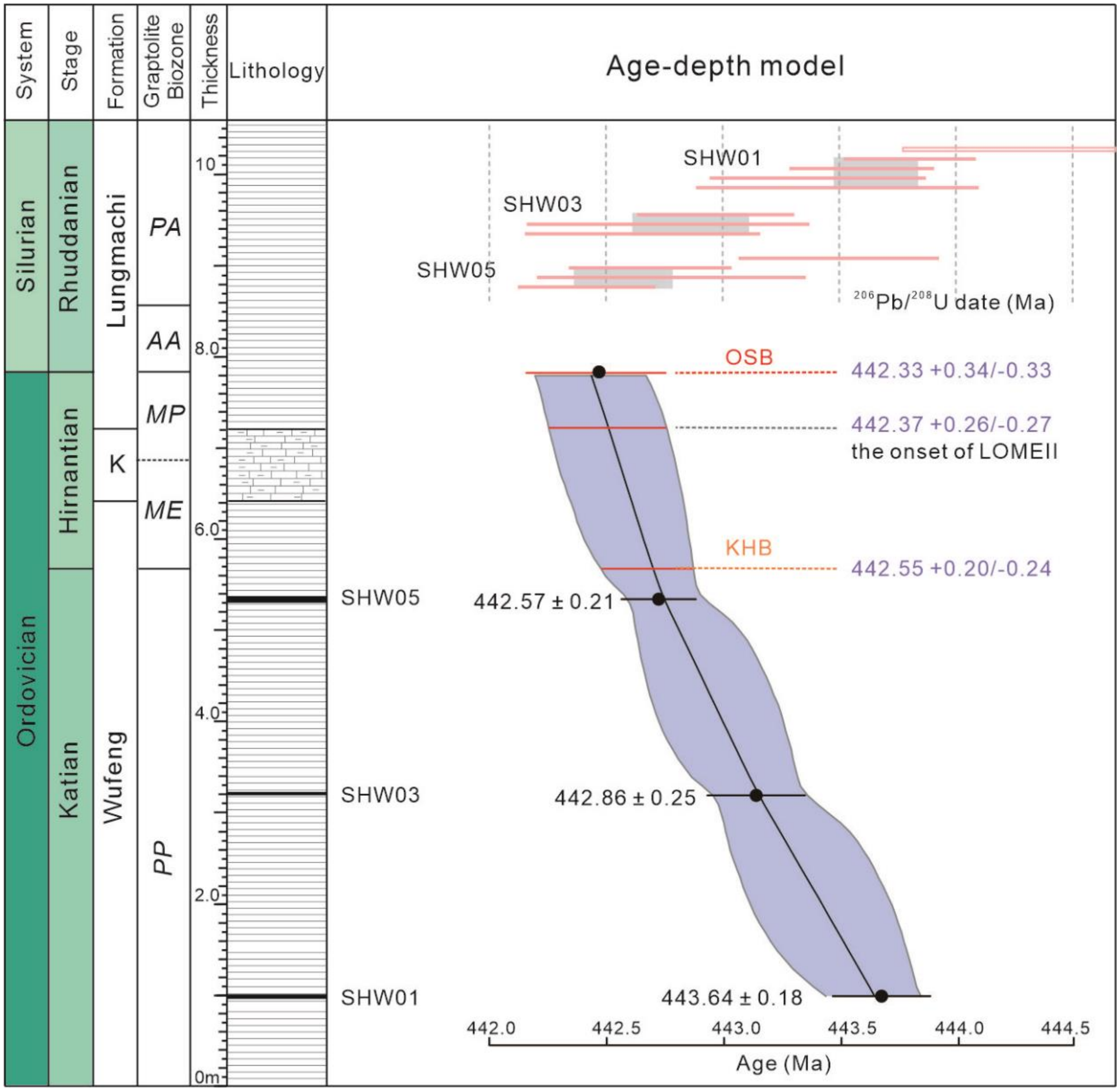

fig. S6. Stratigraphy, geochronology and Bayesian age-depth model for the Shuanghe section. The biostratigraphic calibration is based on ref. (*40*).

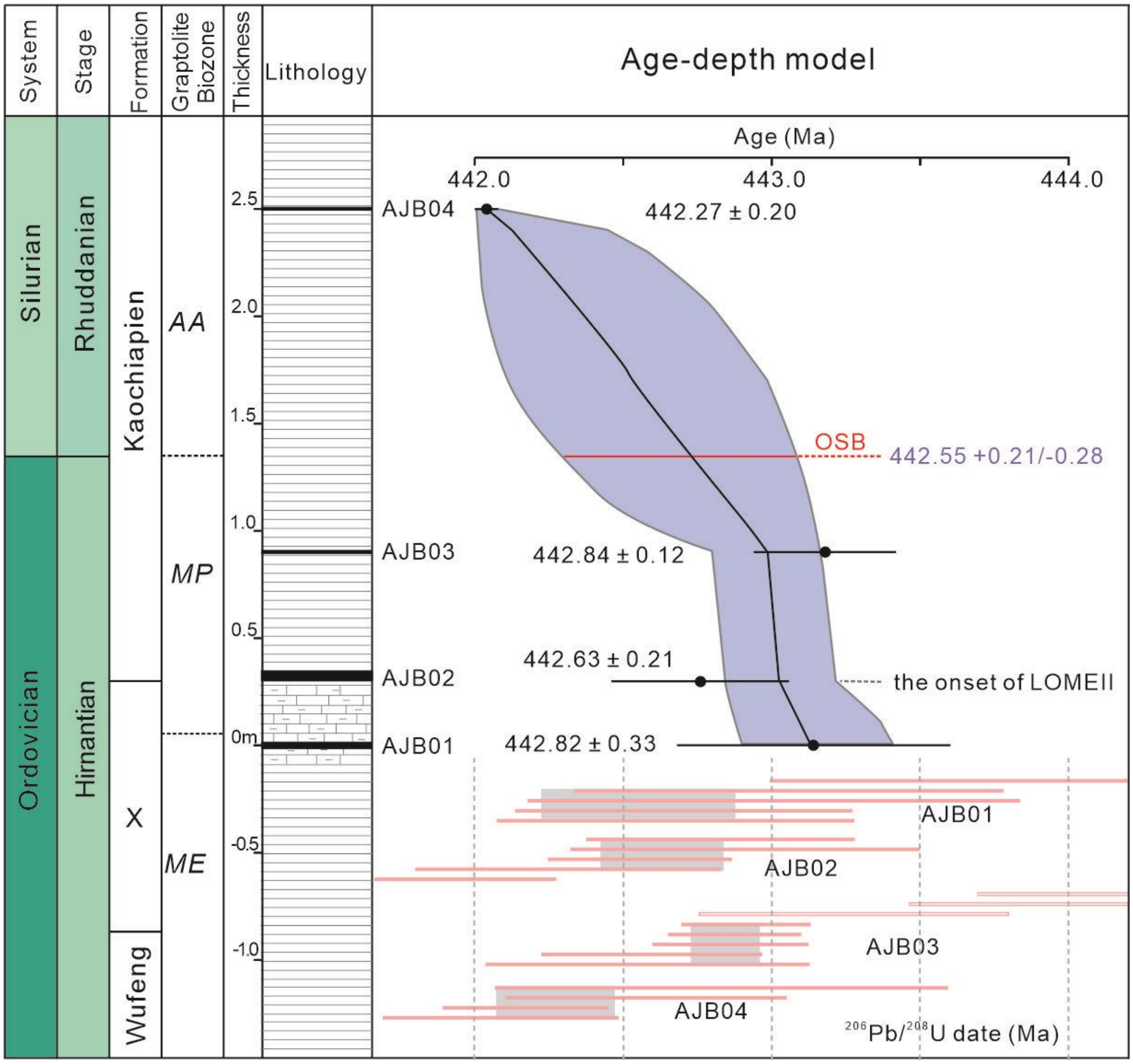

fig. S7. Stratigraphy, geochronology and Bayesian age-depth model for the Beigong section. The biostratigraphic calibration is based on ref. (*39*).

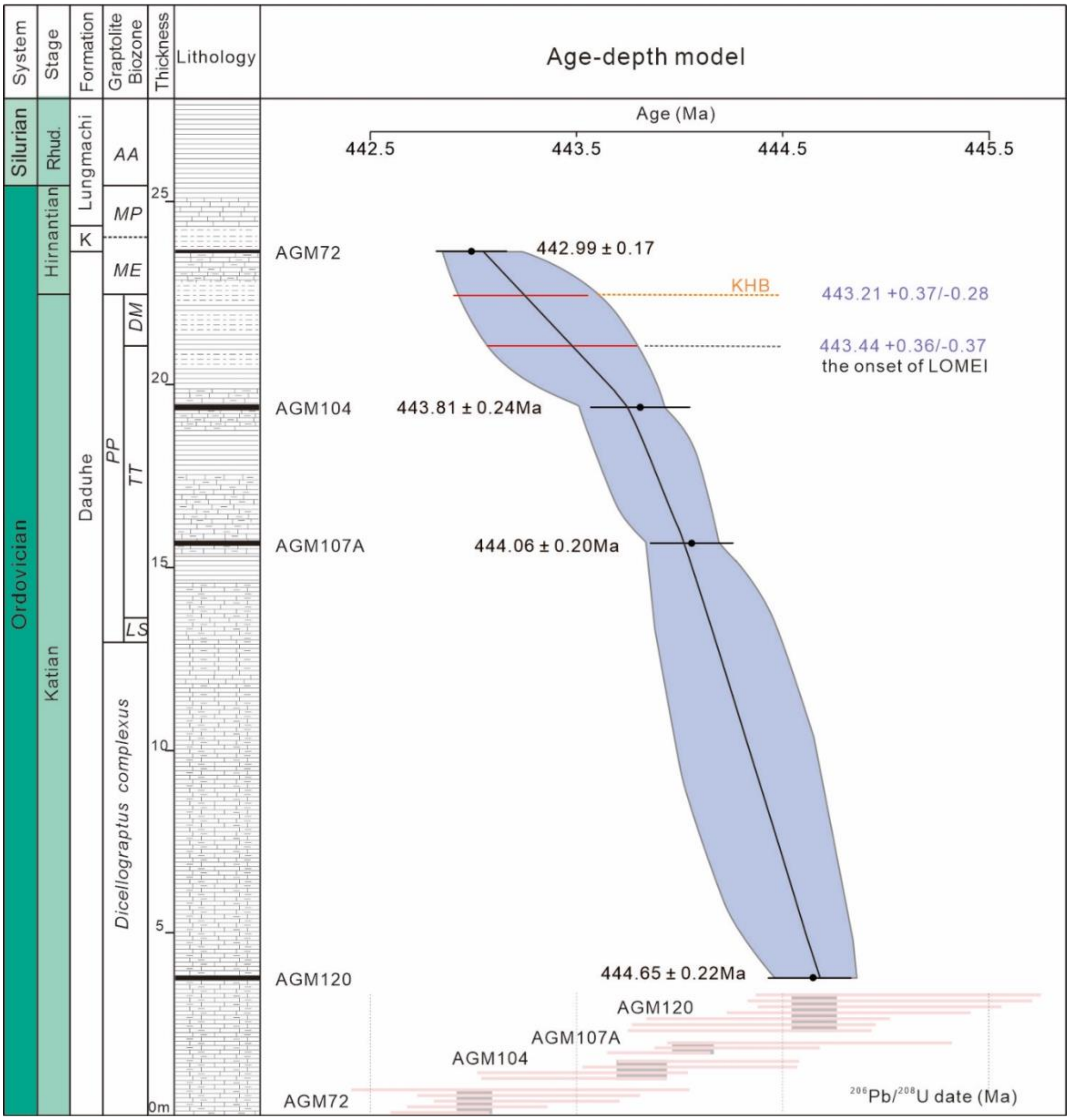

fig. S8. Stratigraphy, geochronology and Bayesian age-depth model for the Wanhe section. $^{206}Pb/^{238}U$ dates are from ref. (*26*).

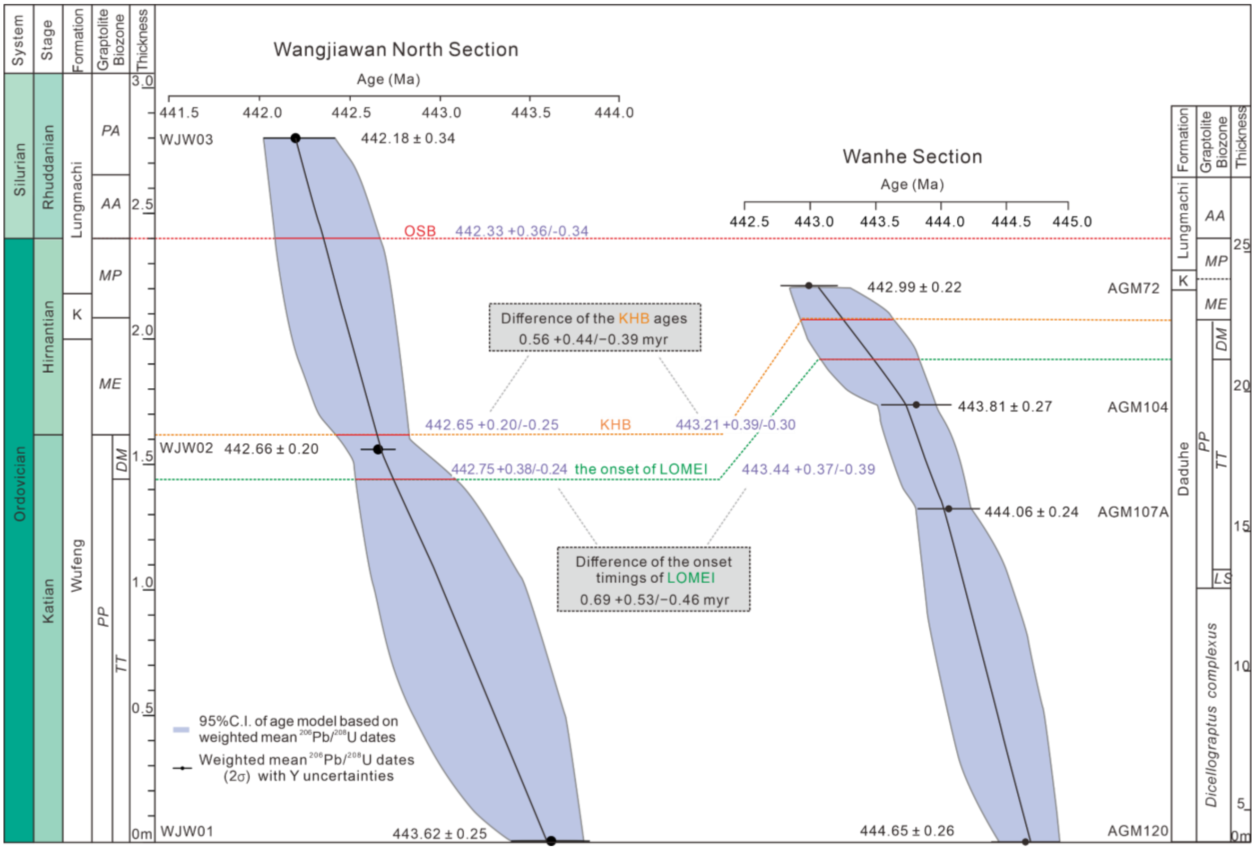

fig. S9. Bayesian age-depth models for Wangjiawan North and Wanhe sections using the dates with Y uncertainties. $^{206}Pb/^{238}U$ dates of Wanhe section are from ref. (*26*).

Table S1 (separate file)
Organic carbon isotope data in Shuanghe section

Table S2 (separate file)
CA-ID-TIMS zircon U-Pb dating results and age interpretations (including published data)

Table S3 (separate file)
CA-ID-TIMS zircon U-Pb data

REFERENCES AND NOTES

1. J. J. Sepkoski, "Patterns of Phanerozoic extinction: A perspective from global data bases," in *Global Events and Event Stratigraphy in the Phanerozoic*. O. H. Walliser, Eds. (Springer, Berlin, 1996), pp. 35–51.

2. D. A. Harper, Late Ordovician mass extinction: Earth, fire and ice. *Natl. Sci. Rev.* **11**, nwad319 (2024).

3. D. A. Harper, E. U. Hammarlund, C. M. Rasmussen, End Ordovician extinctions: A coincidence of causes. *Gondw. Res.* **25**, 1294–1307 (2014).

4. P. M. Sheehan, The Late Ordovician mass extinction. *Annu. Rev. Earth Planet. Sci.* **29**, 331–364 (2001).

5. D. M. Raup, J. J. Sepkoski Jr., Mass extinctions in the marine fossil record. *Science* **215**, 1501–1503 (1982).

6. Y. Deng, J. Fan, S. Zhang, X. Fang, Z. Chen, Y. Shi, H. Wang, X. Wang, J. Yang, X. Hou, Y. Wang, Y. Zhang, Q. Chen, A. Yang, R. Fan, S. Dong, H. Xu, S. Shen, Timing and patterns of the great Ordovician biodiversification event and Late Ordovician mass extinction: Perspectives from South China. *Earth Sci. Rev.* **220**, 103743 (2021).

7. O. E. Sutcliffe, J. A. Dowdeswell, R. J. Whittington, J. N. Theron, J. J. G. Craig, Calibrating the late Ordovician glaciation and mass extinction by the eccentricity cycles of Earth's orbit. *Geology* **28**, 967–970 (2000).

8. P. Brenchley, J. Marshall, C. J. Underwood, Do all mass extinctions represent an ecological crisis? Evidence from the Late Ordovician. *Geol. J.* **36**, 329–340 (2001).

9. S. Finnegan, K. Bergmann, J. M. Eiler, D. S. Jones, D. A. Fike, I. Eisenman, N. C. Hughes, A. K. Tripati, W. W. Fischer, The magnitude and duration of Late Ordovician–Early Silurian glaciation. *Science* **331**, 903–906 (2011).

10. J.-F. Ghienne, A. Desrochers, T. R. Vandenbroucke, A. Achab, E. Asselin, M.-P. Dabard, C. Farley, A. Loi, F. Paris, S. Wickson, J. Veizer, A Cenozoic-style scenario for the end-Ordovician glaciation. *Nat. Commun.* **5**, 4485 (2014).

11. S. Yang, W. Hu, X. Wang, B. Jiang, S. Yao, F. Sun, Z. Huang, F. Zhu, Duration, evolution, and implications of volcanic activity across the Ordovician–Silurian transition in the Lower Yangtze region, South China. *Earth Planet. Sci. Lett.* **518**, 13–25 (2019).

12. D. P. Bond, S. E. Grasby, Late Ordovician mass extinction caused by volcanism, warming, and anoxia, not cooling and glaciation. *Geology* **48**, 777–781 (2020).

13. J. Longman, B. J. Mills, H. R. Manners, T. M. Gernon, M. R. Palmer, Late Ordovician climate change and extinctions driven by elevated volcanic nutrient supply. *Nat. Geosci.* **14**, 924–929 (2021).

14. M. Liu, D. Chen, L. Jiang, R. G. Stockey, D. Aseel, B. Zhang, K. Liu, X. Yang, D. Yan, N. J. Planavsky, Oceanic anoxia and extinction in the latest Ordovician. *Earth Planet. Sci. Lett.* **588**, 117553 (2022).

15. C. Zou, Z. Qiu, S. W. Poulton, D. Dong, H. Wang, D. Chen, B. Lu, Z. Shi, H. Tao, Ocean euxinia and climate change "double whammy" drove the Late Ordovician mass extinction. *Geology* **46**, 535–538 (2018).

16. X. Jing, Z. Yang, R. N. Mitchell, Y. Tong, M. Zhu, B. Wan, Ordovician–Silurian true polar wander as a mechanism for severe glaciation and mass extinction. *Nat. Commun.* **13**, 7941 (2022).

17. T. J. Crowley, G. R. North, Abrupt climate change and extinction events in earth history. *Science* **240**, 996–1002 (1988).

18. S. Finnegan, N. A. Heim, S. E. Peters, W. W. Fischer, Climate change and the selective signature of the Late Ordovician mass extinction. *Proc. Natl. Acad. Sci. U.S.A.* **109**, 6829–6834 (2012).

19. P. J. Mayhew, M. A. Bell, T. G. Benton, A. J. McGowan, Biodiversity tracks temperature over time. *Proc. Natl. Acad. Sci. U.S.A.* **109**, 15141–15145 (2012).

20. S. L. Goldberg, T. M. Present, S. Finnegan, K. D. Bergmann, A high-resolution record of early Paleozoic climate. *Proc. Natl. Acad. Sci. U.S.A.* **118**, e2013083118 (2021).

21. H. Song, D. B. Kemp, L. Tian, D. Chu, H. Song, X. Dai, Thresholds of temperature change for mass extinctions. *Nat. Commun.* **12**, 4694 (2021).

22. S. Shen, F. Zhang, W. Wang, J. Fan, J. Chen, B. Wang, J. Cao, S. Yang, H. Zhang, G. Li, T. Deng, X. Li, J. Chen, Deep-time major biological and climatic events versus global changes: Progresses and challenges. *Sci. Bull.* **69**, 268–285 (2024).

23. Y. Hu, J. Zhou, B. Song, W. Li, W. Sun, SHRIMP zircon U-Pb dating from K-bentonite in the top of Ordovician of Wangjiawan Section, Yichang, Hubei, China. *Sci. China Ser. D Earth Sci.* **51**, 493–498 (2008).

24. R. Tucker, T. Krogh, R. Ross Jr., S. Williams, Time-scale calibration by high-precision U–Pb zircon dating of interstratified volcanic ashes in the Ordovician and Lower Silurian stratotypes of Britain. *Earth Planet. Sci. Lett.* **100**, 51–58 (1990).

25. Y. Zhong, H. Wu, J. Fan, Q. Fang, M. Shi, S. Zhang, T. Yang, H. Li, L. Cao, Late Ordovician obliquity-forced glacio-eustasy recorded in the Yangtze Block, South China. *Palaeogeogr. Palaeoclimatol. Palaeoecol.* **540**, 109520 (2020).

26. M. Ling, R. Zhan, G. Wang, Y. Wang, Y. Amelin, P. Tang, J. Liu, J. Jin, B. Huang, R. Wu, S. Xue, B. Fu, V. C. Bennett, X. Wei, X. C. Luan, S. Finnegan, D. A. T. Harper, J. Y. Rong, An extremely brief end Ordovician mass extinction linked to abrupt onset of glaciation. *Solid Earth Sci.* **4**, 190–198 (2019).

27. D. Goldman, P. M. Sadler, S. A. Leslie, M. J. Melchin, F. P. Agterberg, F. M. Gradstein, "The Ordovician period," in *Geologic Time Scale 2020*, F. M. Gradstein, J. G. Ogg, M. D. Schmitz, G. M. Ogg, Eds. (Elsevier, 2020), pp. 631–694.

28. X. Chen, M. J. Melchin, H. D. Sheets, C. E. Mitchell, F. Jun-Xuan, Patterns and processes of latest Ordovician graptolite extinction and recovery based on data from South China. *J. Paleo.* **79**, 842–861 (2005).

29. J. Rong, D. A. T. Harper, B. Huang, R. Li, X. Zhang, D. Chen, The latest Ordovician Hirnantian brachiopod faunas: New global insights. *Earth Sci. Rev.* **208**, 103280 (2020).

30. J. Rong, D. A. Harper, A global synthesis of the latest Ordovician Hirnantian brachiopod faunas. *Earth Environ. Sci. Trans. R. Soc. Edinb.* **79**, 383–402 (1988).

31. J. Rong, D. A. Harper, Brachiopod survival and recovery from the latest Ordovician mass extinctions in South China. *Geol. J.* **34**, 321–348 (1999).

32. P. J. Brenchley, G. A. Carden, L. Hints, D. Kaljo, J. D. Marshall, T. Martma, T. Meidla, J. Nõlvak, High-resolution stable isotope stratigraphy of Upper Ordovician sequences: Constraints on the timing of bioevents and environmental changes associated with mass extinction and glaciation. *Geol. Soc. Am. Bull.* **115**, 89–104 (2003).

33. C. M. O. Rasmussen, B. Kroger, M. L. Nielsen, J. Colmenar, Cascading trend of Early Paleozoic marine radiations paused by Late Ordovician extinctions. *Proc. Natl. Acad. Sci. U.S.A.* **116**, 7207–7213 (2019).

34. G. Wang, R. Zhan, I. G. Percival, The end-Ordovician mass extinction: A single-pulse event? *Earth Sci. Rev.* **192**, 15–33 (2019).

35. X. Chen, J. Rong, J. Fan, R. Zhan, C. E. Mitchell, D. A. T. Harper, M. J. Melchin, S. C. Finney, X. Wang, The Global Boundary Stratotype Section and Point (GSSP) for the base of the Hirnantian Stage (the uppermost of the Ordovician System). *J. Int. Geosci.* **29**, 183–196 (2006).

36. B. Schoene, M. P. Eddy, K. M. Samperton, C. B. Keller, G. Keller, T. Adatte, S. F. R. Khadri, U-Pb constraints on pulsed eruption of the deccan traps across the end-Cretaceous mass extinction. *Science* **363**, 862–866 (2019).

37. P. J. Brenchley, J. D. Marshall, G. A. F. Carden, D. B. R. Robertson, D. G. F. Long, T. Meidla, L. Hints, T. F. Anderson, Bathymetric and isotopic evidence for a short-lived Late Ordovician glaciation in a greenhouse period. *Geology* **22**, 295–298 (1994).

38. E. E. Saupe, H. Qiao, Y. Donnadieu, A. Farnsworth, A. T. Kennedy-Asser, J. B. Ladant, D. J. Lunt, A. Pohl, P. Valdes, S. Finnegan, Extinction intensity during Ordovician and Cenozoic glaciations explained by cooling and palaeogeography. *Nat. Geosci.* **13**, 65–70 (2020).

39. J. Rong, X. Chen, D. A. T. Harper, The latest Ordovician Hirnantia Fauna (Brachiopoda) in time and space. *Lethaia* **35**, 231–249 (2002).

40. W. Duan, "Graptolite biostratigraphy and carbon isotope stratigraphy of the Upper Ordovician-Lower Silurian in Changning, Sichuan, China," thesis, China Univ. of Geosciences, Beijing (2011).

41. J. Rong, B. Huang, The first brachiopod fauna following Late Ordovician mass extinction: Evidence from late Hirnantian brachiopods of Zhenxiong, Yunnan, SW China. *Acta Palaeontol. Sin.* **62**, 1–29 (2023).

42. B. Huang, Y. Candela, K. Shi, J. Rong, A new post-LOME (Late Ordovician mass extinction) recovery brachiopod fauna from South China. *J. Paleo.* **98**, 366–377 (2024).

43. A.-C. Da Silva, M. Sinnesael, P. Claeys, J. H. F. L. Davies, N. J. de Winter, L. M. E. Percival, U. Schaltegger, D. De Vleeschouwer, Anchoring the Late Devonian mass extinction in absolute time by integrating climatic controls and radio-isotopic dating. *Sci. Rep.* **10**, 12940 (2020).

44. S. D. Burgess, S. Bowring, S. Shen, High-precision timeline for Earth's most severe extinction. *Proc. Natl. Acad. Sci. U.S.A.* **111**, 3316–3321 (2014).

45. B. Schoene, J. Guex, A. Bartolini, U. Schaltegger, T. J. Blackburn, Correlating the end-Triassic mass extinction and flood basalt volcanism at the 100 ka level. *Geology* **38**, 387–390 (2010).

46. L. W. Alvarez, W. Alvarez, F. Asaro, H. V. Michel, Extraterrestrial cause for the Cretaceous-Tertiary extinction. *Science* **208**, 1095–1108 (1980).

47. S. L. Kamo, G. K. Czamanske, Y. Amelin, V. A. Fedorenko, D. W. Davis, V. R. Trofimov, Rapid eruption of Siberian flood-volcanic rocks and evidence for coincidence with the Permian–Triassic boundary and mass extinction at 251 Ma. *Earth Planet. Sci. Lett.* **214**, 75–91 (2003).

48. U. Schaltegger, J. Guex, A. Bartolini, B. Schoene, M. Ovtcharova, Precise U-Pb age constraints for end-Triassic mass extinction, its correlation to volcanism and Hettangian post-extinction recovery. *Earth Planet. Sci. Lett.* **267**, 266–275 (2008).

49. D. P. Bond, S. E. Grasby, On the causes of mass extinctions. *Palaeogeogr. Palaeoclimatol. Palaeoecol.* **478**, 3–29 (2017).

50. T. J. Blackburn, P. E. Olsen, S. A. Bowring, N. M. McLean, D. V. Kent, J. Puffer, G. McHone, E. T. Rasbury, M. Et-Touhami, Zircon U-Pb geochronology links the end-Triassic extinction with the central atlantic magmatic province. *Science* **340**, 941–945 (2013).

51. S. D. Burgess, S. A. Bowring, High-precision geochronology confirms voluminous magmatism before, during, and after Earth's most severe extinction. *Sci. Adv.* **1**, e1500470 (2015).

52. K. R. Chamberlain, A. K. Khudoley, R. E. Ernst, A. V. Prokopiev, Improved U–Pb dating of the ca. 450 Ma Suordakh mafic event in eastern Siberia will test whether this is the missing LIP related to end–Ordovician mass extinction: Progress report. Abstract presented at the Large igneous provinces through earth history: Mantle plumes, supercontinents, climate change, metallogeny and oil-gas, planetary analogues (Tomsk, Russia, 2019), pp. 9–11.

53. A. K. Khudoley, A. V. Prokopiev, K. R. Chamberlain, A. D. Savelev, R. E. Ernst, S. V. Malyshev, A. N. Moskalenko, O. Y. Lebedeva, Late ordovician mafic magmatic event, Southeast Siberia: Tectonic implications, LIP interpretation, and potential link with a mass extinction. *Minerals* **10**, 1108 (2020).

54. R. E. Ernst, D. P. G. Bond, S.-H. Zhang, K. L. Buchan, S. E. Grasby, N. Youbi, H. El Bilali, A. Bekker, L. S. Doucet, “Large igneous province record through time and implications for secular environmental changes and geological time-scale boundaries,” in *Large Igneous Provinces: A Driver of Global Environmental Biotic Changes*, R. E. Ernst, A. Dickson J, A. Bekker, Eds. (2021), pp. 1–26.

55. M. Derakhshi, R. E. Ernst, S. L. Kamo, Ordovician-Silurian volcanism in northern Iran: Implications for a new Large Igneous Province (LIP) and a robust candidate for the Late Ordovician mass extinction. *Gondw. Res.* **107**, 256–280 (2022).

56. J. A. Trotter, I. S. Williams, C. R. Barnes, C. Lécuyer, R. S. Nicoll, Did cooling oceans trigger Ordovician biodiversification? Evidence from conodont thermometry. *Science* **321**, 550–554 (2008).

57. N. Thiagarajan, A. Lepland, U. Ryb, T. H. Torsvik, L. Ainsaar, O. Hints, J. Eiler, Reconstruction of Phanerozoic climate using carbonate clumped isotopes and implications for the oxygen isotopic composition of seawater. *Proc. Natl. Acad. Sci. U.S.A.* **121**, e2400434121 (2024).

58. A. Pohl, E. Nardin, T. R. Vandenbroucke, Y. Donnadieu, “The Ordovician ocean circulation: A modern synthesis based on data and models,” in *A Global Synthesis of the Ordovician System* (Geological Society of London, 2023), vol. 532, pp. 157–169.

59. G. T. Pecl, M. B. Araújo, J. D. Bell, J. Blanchard, T. C. Bonebrake, I.-C. Chen, T. D. Clark, R. K. Colwell, F. Danielsen, B. Evengård, L. Falconi, S. Ferrier, S. Frusher, R. A. Garcia, R. B. Griffis, A. J. Hobday, C. Janion-Scheepers, M. A. Jarzyna, S. Jennings, J. Lenoir, H. I. Linnetved, V. Y. Martin, P. C. McCormack, J. McDonald, N. J. Mitchell, T. Mustonen, J. M. Pandolfi, N. Pettorelli, E. Popova, S. A. Robinson, B. R. Scheffers, J. D. Shaw, C. J. B. Sorte, J. M. Strugnell, J. M. Sunday, M. N. Tuanmu, A. Vergés, C. Villanueva, T. Wernberg, E. Wapstra, S. E. Williams, Biodiversity redistribution under climate change: Impacts on ecosystems and human well-being. *Science* **355**, eaai9214 (2017).

60. D. J. Condon, B. Schoene, N. M. McLean, S. A. Bowring, R. R. Parrish, Metrology and traceability of U-Pb isotope dilution geochronology (EARTHTIME Tracer Calibration Part I). *Geochim. Cosmochim. Acta* **164**, 464–480 (2015).

61. N. M. McLean, D. J. Condon, B. Schoene, S. A. Bowring, Evaluating uncertainties in the calibration of isotopic reference materials and multi-element isotopic tracers (EARTHTIME Tracer Calibration Part II). *Geochim. Cosmochim. Acta* **164**, 481–501 (2015).

62. J. Hiess, D. J. Condon, N. McLean, S. R. Noble, $^{238}U/^{235}U$ systematics in terrestrial uranium-bearing minerals. *Science* **335**, 1610–1614 (2012).

63. M. D. Schmitz, B. Schoene, Derivation of isotope ratios, errors, and error correlations for U-Pb geochronology using ^{205}Pb-^{235}U-(^{233}U)-spiked isotope dilution thermal ionization mass spectrometric data. *Geochem. Geophys. Geosyst.* **8**, Q08006 (2007).

64. N. M. McLean, J. F. Bowring, S. A. Bowring, An algorithm for U-Pb isotope dilution data reduction and uncertainty propagation. *Geochem. Geophys. Geosyst.* **12**, Q0AA18 (2011).

65. J. F. Bowring, N. M. McLean, S. Bowring, Engineering cyber infrastructure for U-Pb geochronology: Tripoli and U-Pb_Redux. *Geochem. Geophys. Geosyst.* **12**, Q0AA19 (2011).

66. D. Condon, B. Schoene, M. Schmitz, U. Schaltegger, R. B. Ickert, Y. Amelin, L. E. Augland, K. R. Chamberlain, D. S. Coleman, J. N. Connelly, F. Corfu, J. L. Crowley, J. H. F. L. Davies, S. W. Denyszyn, M. P. Eddy, S. P. Gaynor, L. M. Heaman, M. H. Huyskens, S. Kamo, J. Kasbohm, C. B. Keller, S. A. MacLennan, N. M. McLean, S. Noble, M. Ovtcharova, A. Paul, J. Ramezani, M. Rioux, D. Sahy, J. S. Scoates, D. Szymanowski, S. Tapster, M. Tichomirowa, C. J. Wall, J.-F. Wotzlaw, C. Yang, Q.-Z. Yin, Recommendations for the reporting and interpretation of isotope dilution U-Pb geochronological information. *Geol. Soc. Am. Bull.* **136**, 4233–4251 (2024).

67. M. H. Huyskens, S. Zink, Y. Amelin, Evaluation of temperature-time conditions for the chemical abrasion treatment of single zircons for U-Pb geochronology. *Chem. Geol.* **438**, 25–35 (2016).

68. A. Jaffey, K. Flynn, L. Glendenin, W. T. Bentley, A. Essling, Precision measurement of half-lives and specific activities of ^{235}U and ^{238}U. *Phys. Rev. C* **4**, 1889–1906 (1971).

69. T. H. Torsvik, J. G. Meert, M. Domeier, Ordovician palaeogeography and climate change. *Gondwana Res.* **100**, 63–95 (2020).

70. B. Huang, J. Rong, Heterogeneous palaeo-ecogeography of brachiopods during the Late Ordovician mass extinction in South China. *Palaeontology* **67**, e12728 (2024).

71. L. Li, H. Feng, D. Janussen, J. Reitner, Unusual Deep Water sponge assemblage in South China—Witness of the end-Ordovician mass extinction. *Sci. Rep.* **5**, 16060 (2015).

72. J. Zhang, C. Li, Y. Zhong, X. Wu, X. Fang, M. Liu, D. Chen, B. C. Gill, T. J. Algeo, T. W. Lyons, Y. Zhang, H. Tian, Linking carbon cycle perturbations to the Late Ordovician glaciation and mass extinction: A modeling approach. *Earth Planet. Sci. Lett.* **631**, 118635 (2024).

73. P. Gorjan, K. Kaiho, D. A. Fike, C. Xu, Carbon-and sulfur-isotope geochemistry of the Hirnantian (Late Ordovician) Wangjiawan (Riverside) section, South China: Global correlation and environmental event interpretation. *Palaeogeogr. Palaeoclimatol. Palaeoecol.* **337-338**, 14–22 (2012).

74. J. Wang, Z. Li, History of Neoproterozoic rift basins in South China: Implications for Rodinia break-up. *Precambrian Res.* **122**, 141–158 (2003).

75. X. Chen, J. Rong, X. Wang, Z. Wang, Y. Zhang, R. Zhan, in *Correlation of the Ordovician rocks of China: Charts and Explanatory Notes*. (International Union of Geological Sciences, 1995), vol. 31, pp. 1–104.

76. R. Zhan, J. Jin, in *Ordovician–Early Silurian (Llandovery) Stratigraphy and Palaeontology of the Upper Yangtze Platform*, *South China* (Science Press, 2007), pp. 1–169.

77. C. Yang, M. Zhu, D. J. Condon, X. Li, Geochronological constraints on stratigraphic correlation and oceanic oxygenation in Ediacaran-Cambrian transition in South China. *J. Asian Earth Sci.* **140**, 75–81 (2017).

78. L. R. Cocks, R. A. Fortey, Lower Palaeozoic facies and faunas around Gondwana. *Geol. Soc. Lond. Spec. Publ.* **37**, 183–200 (1988).

79. R. A. Fortey, L. R. M. Cocks, Palaeontological evidence bearing on global Ordovician–Silurian continental reconstructions. *Earth Sci. Rev.* **61**, 245–307 (2003).

80. L. R. M. Cocks, T. H. Torsvik, Baltica from the late Precambrian to mid-Palaeozoic times: The gain and loss of a terrane's identity. *Earth Sci. Rev.* **72**, 39–66 (2005).

81. J. Jin, R. Zhan, R. Wu, Equatorial cold-water tongue in the Late Ordovician. *Geology* **46**, 759–762 (2018).

82. P. J. Brenchley, G. Newall, A facies analysis of Upper Ordovician regressive sequences in the Oslo region, Norway—A record of glacio-eustatic changes. *Palaeogeogr. Palaeoclimatol. Palaeoecol.* **31**, 1–38 (1980).

83. D. G. Long, Oxygen and carbon isotopes and event stratigraphy near the Ordovician—Silurian boundary, Anticosti Island Quebec. *Palaeogeogr. Palaeoclimatol. Palaeoecol.* **104**, 49–59 (1993).

84. S. Zhang, C. R. Barnes, A new Llandovery (early Silurian) conodont biozonation and conodonts from the Becscie, Merrimack, and Gun River formations, Anticosti Island, Québec. *J. Paleontol.* **76**, 1–46 (2002).

85. X. Chen, J. Rong, Y. Li, A. J. Boucot, Facies patterns and geography of the Yangtze region, South China, through the Ordovician and Silurian transition. *Palaeogeogr. Palaeoclimatol. Palaeoecol.* **204**, 353–372 (2004).

86. M. J. Melchin, C. E. Mitchell, C. Holmden, P. Štorch, Environmental changes in the Late Ordovician–early Silurian: Review and new insights from black shales and nitrogen isotopes. *Geol. Soc. Am. Bull.* **125**, 1635–1670 (2013).

87. E. Mu, J. Li, M. Ge, X. Chen, Y. Lin, Y. Ni, "Upper Ordovician graptolites of central China region," in Palaeontologia Sinica: Series B (Science Press, 1993), vol. 182, pp. 1–393.

88. W. Wang, W. Hu, Q. Chen, D. Jia, X. Chen, Temporal and spatial distribution of Ordovician–Silurian boundary black graptolitic shales on the Lower Yangtze Platform. *Palaeoworld* **26**, 444–455 (2017).

89. J. Rong, The Hirnantia fauna of China with comments on the Ordovician-Silurian boundary. *Acta Stratigr. Sin.* **3**, 1–29 (1979).

90. P. Tang, B. Huang, R. Wu, J. Fan, K. Yan, G. Wang, J. Liu, Y. Wang, R. Zhan, J. Rong, On the upper Ordovician Daduhe Formation of the upper Yangtze region. *J. Stratig.* **41**, 119–133 (2017).
